# Structures of human telomerase with BIBR1532 reveal novel mechanism of inhibition

**DOI:** 10.1038/s41589-026-02238-6

**Published:** 2026-06-02

**Authors:** Yuqing Wang, Baocheng Liu, Yao He, Juli Feigon

**Affiliations:** 1 Department of Chemistry and Biochemistry, University of California, Los Angeles, Los Angeles, CA USA; 2 Department of Microbiology, Immunology and Molecular Genetics, University of California, Los Angeles, Los Angeles, CA USA

**Keywords:** Enzyme mechanisms, Target identification, Structural biology

## Abstract

Human telomerase processively adds telomeric repeats (dGGTTAG) to chromosome 3′-ends to maintain telomere length. While mostly absent in somatic cells, telomerase is aberrantly upregulated in most tumor cells to sustain cellular immortality, making it a promising oncology target. However, to date there are no reported structures of human telomerase with inhibitor, impeding structure-based drug design and optimization. We report nine cryo-electron microscopy structures of human telomerase with and without BIBR1532, a highly selective small-molecule telomerase inhibitor. Unexpectedly, BIBR1532 binds a previously unknown pocket between TERT finger and palm. BIBR1532 inhibits each step but disproportionately affects the rate-limiting first step of telomere repeat nucleotide addition. The structures reveal a rigid finger that explains telomerase’s slow rate and low fidelity. Our study provides insights into telomerase catalytic mechanism and its inhibition by BIBR1532, explains why prior BIBR derivatives did not improve potency and suggests a rational approach for design of small-molecule telomerase inhibitors.

**Figure:**
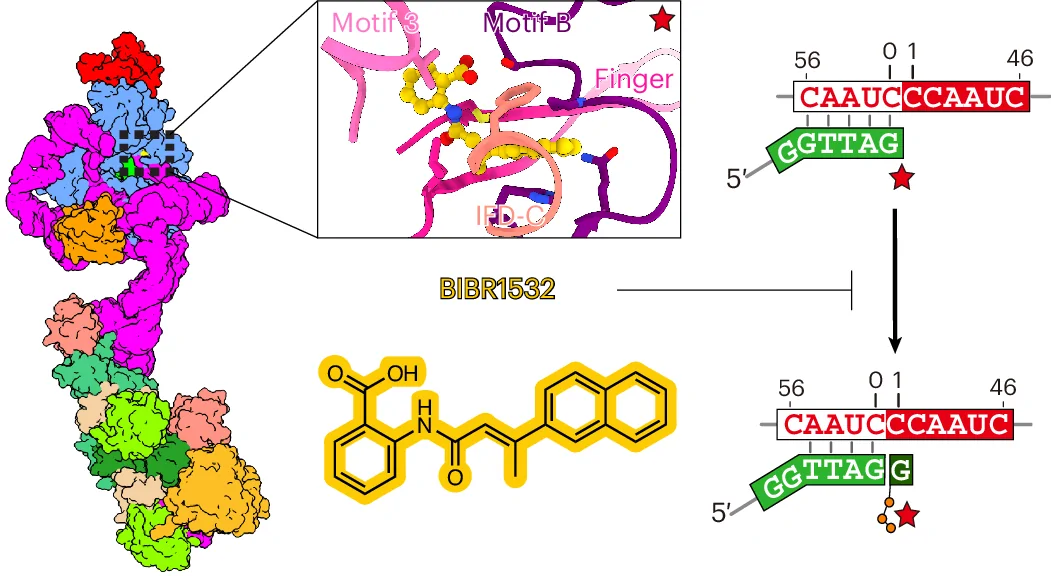

## Abstract (web summary)

Telomerase, the ribonucleoprotein that maintains telomere ends, is upregulated in most cancer cells, making it a promising oncology target. Cryo-electron microscopy structures of telomerase with allosteric inhibitor BIBR1532 reveal an unexpected mode of inhibition and unique insights into telomerase mechanism.

## Main

**Fig. 1:**
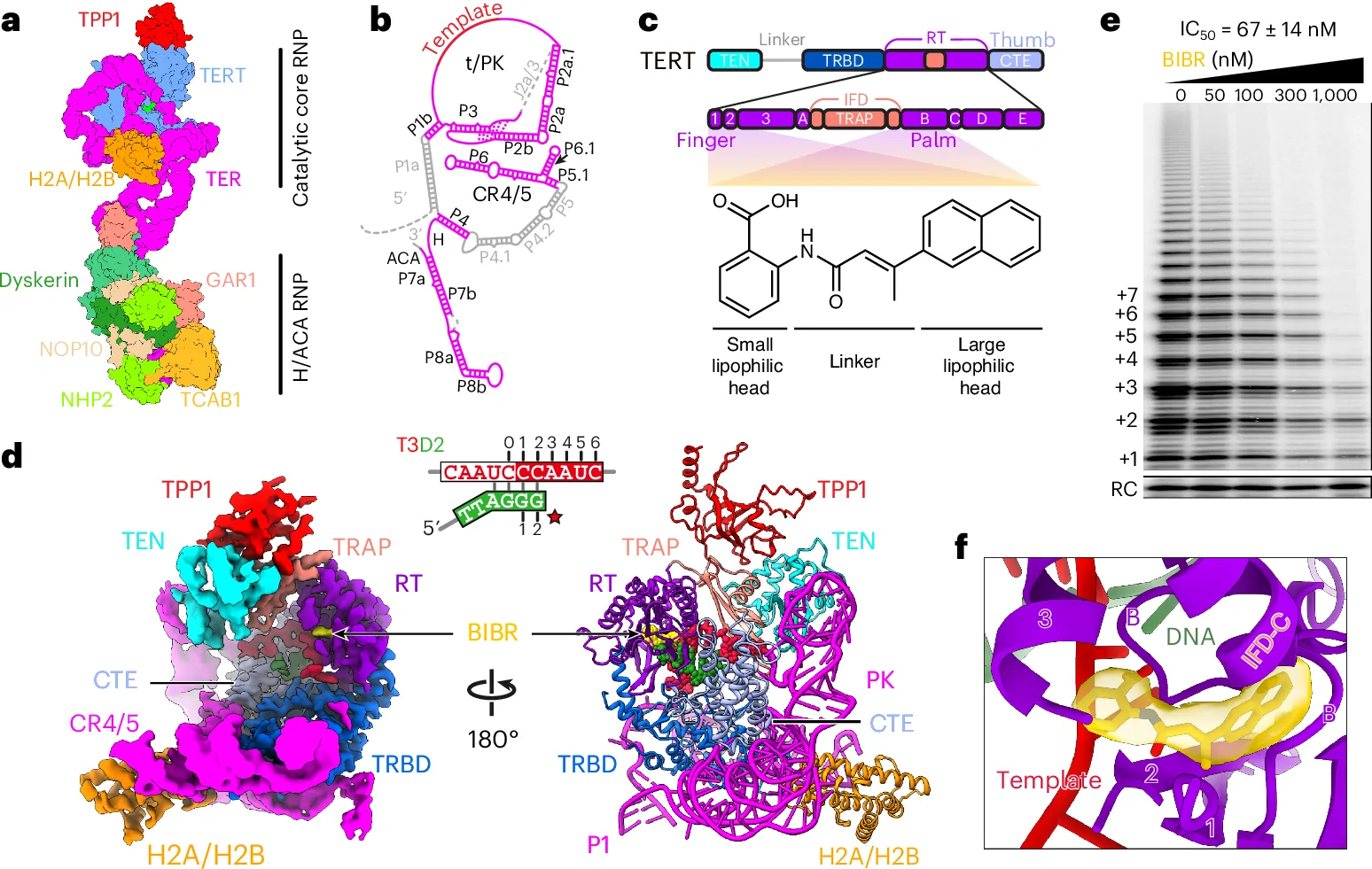
Cryo-EM structure of human telomerase with d(TTAGGG)_3_, TPP1 and BIBR1532. **a**, Overall model (surface view) of human telomerase bound to DNA primer d(TTAGGG)_3_, TPP1 and BIBR1532. **b**, Secondary structure of TER, with regions resolved in the cryo-EM map shown in magenta (or red for template) and unresolved regions in gray. **c**, Top: domain organization of TERT. Middle: specific motif organization of RT domain highlighting the BIBR1532-binding site. Bottom: chemical structure of BIBR1532. **d**, Focused refinement of the catalytic core RNP from **a**, showing a 3.1-Å-resolution cryo-EM density map (left) and corresponding cartoon model, rotated 180° (right). TER and TERT are colored the same as in **b** and **c** and BIBR1532 is colored in gold. Inset, schematic of template–primer DNA duplex, illustrating the four base pairs formed between template and primer. The alignment region of the template is white, the last six nucleotides of the primer are green and the red star indicates the active site. **e**, Direct telomerase activity assay of human telomerase as a function of BIBR1532 concentration. The number of telomeric repeats added to the primer is indicated on the left. A representative result from three biologically independent experiments is shown. **f**, Zoomed-in view of BIBR-binding pocket with density shown for BIBR1532.

Telomeres, the physical ends of eukaryotic chromosomes, comprise double-stranded DNA with a repetitive sequence (in humans, dGGTTAG paired with dCTAACC) ending in a 3′ single-strand dGGTTAG repeat overhang and a set of six associated proteins collectively called shelterin^1–3^. Telomeres have a crucial role in maintaining genomic integrity by protecting chromosome termini from degradation and interchromosomal fusions^2,3^. Telomeres progressively shorten with each round of cell division because of incomplete replication^4^. When telomeres shorten to a critical length, cellular senescence or apoptosis is triggered^5^. This process of telomere erosion is mainly counteracted by telomerase^1^. Human telomerase comprises a 451-nt noncoding telomerase RNA (TER or hTR) that forms a catalytic core ribonucleoprotein (RNP) with telomerase reverse transcriptase (TERT) and a histone H2A/H2B dimer and a biogenesis H/ACA RNP with two sets of H/ACA proteins plus TCAB1 that are flexibly linked through TER, as seen in cryo-electron microscopy (cryo-EM) structures^6–9^ (Fig. 1a–c). Telomerase uses its unique template within TER (Fig. 1b,d) to add multiple telomeric repeats to the 3′-overhang after DNA replication^1^. In higher eukaryotes, telomerase activity is restricted to highly proliferative cells such as germline cells and stem cells and is undetectable in most somatic cells^5^. However, telomerase is aberrantly upregulated in most (~90%) human cancers to overcome a cascade of DNA damage responses at critically short telomeres to maintain tumorigenesis, making it a promising target for anticancer drug development^5^.

Extensive efforts over decades have been made to develop telomerase inhibitors^5^. These include oligonucleotide-based inhibitors such as imetelstat (GRN163L), which was recently approved in the US as the first telomerase-targeting anticancer drug, for treating lower-risk myelodysplastic syndrome^10^, nucleoside and nucleotide analogs, such as 6-thioguanine, which selectively inhibits telomerase nucleotide addition and has progressed to a phase 2 clinical trial^11^, indirect inhibitors, such as the G-quadruplex stabilizer BRACO19, which targets the promoter region of TERT^5^, and small molecules developed to directly inhibit telomerase catalytic activity^5^. Among the latter, BIBR1532 (2-[(*E*)-3-naphthalen-2-yl-but-2-enoylamino]-benzoic acid) (Fig. 1c) has emerged as a highly promising direct inhibitor because of its specificity for human telomerase^12^. Widely used in telomere and telomerase research, BIBR1532 selectively inhibits cancer cell proliferation while sparing normal cells^13–15^. However, its half-maximal inhibitory concentration (IC_50_) of ~100 nM in vitro^12^ and ~1 μM in cells^15^ and its poor bioavailability have limited its therapeutic utility^5^. Despite this, BIBR1532 remains a valuable lead compound for structure–activity relationship (SAR) drug development. A crystal structure of the evolutionarily distant *Tribolium* *castaneum* TERT (*Tc*TERT), which lacks an identified TER^16^, showed that BIBR1532 binds *Tc*TERT on the C-terminal extension (CTE) domain, on the basis of which a homologous binding site on human TERT was computationally inferred^16^. However, SAR efforts based on this model have so far failed to produce derivatives more effective than the original BIBR1532 (refs. ^17–20^).

Here we present nine cryo-EM structures of human telomerase, at 2.7–3.4-Å resolution for the catalytic core RNP, that capture different stages of nucleotide addition without and with BIBR1532. The structures and functional studies reveal an unexpected binding pocket in the finger/palm of TERT away from the active site but within a previously unknown network of interactions important for catalysis. These studies not only explain the mechanism of telomerase inhibition by BIBR1532 but also provide unique insights into telomerase mechanism, including its slow overall rate^21^, low fidelity^22^ and rate-limiting first step^23^. Furthermore, they suggest a pathway for developing BIBR inhibitors with improved telomerase binding and inhibition for therapeutic applications.

## Results

### Identification of the BIBR1532-binding pocket on telomerase

**Extended Data Fig. 1:**
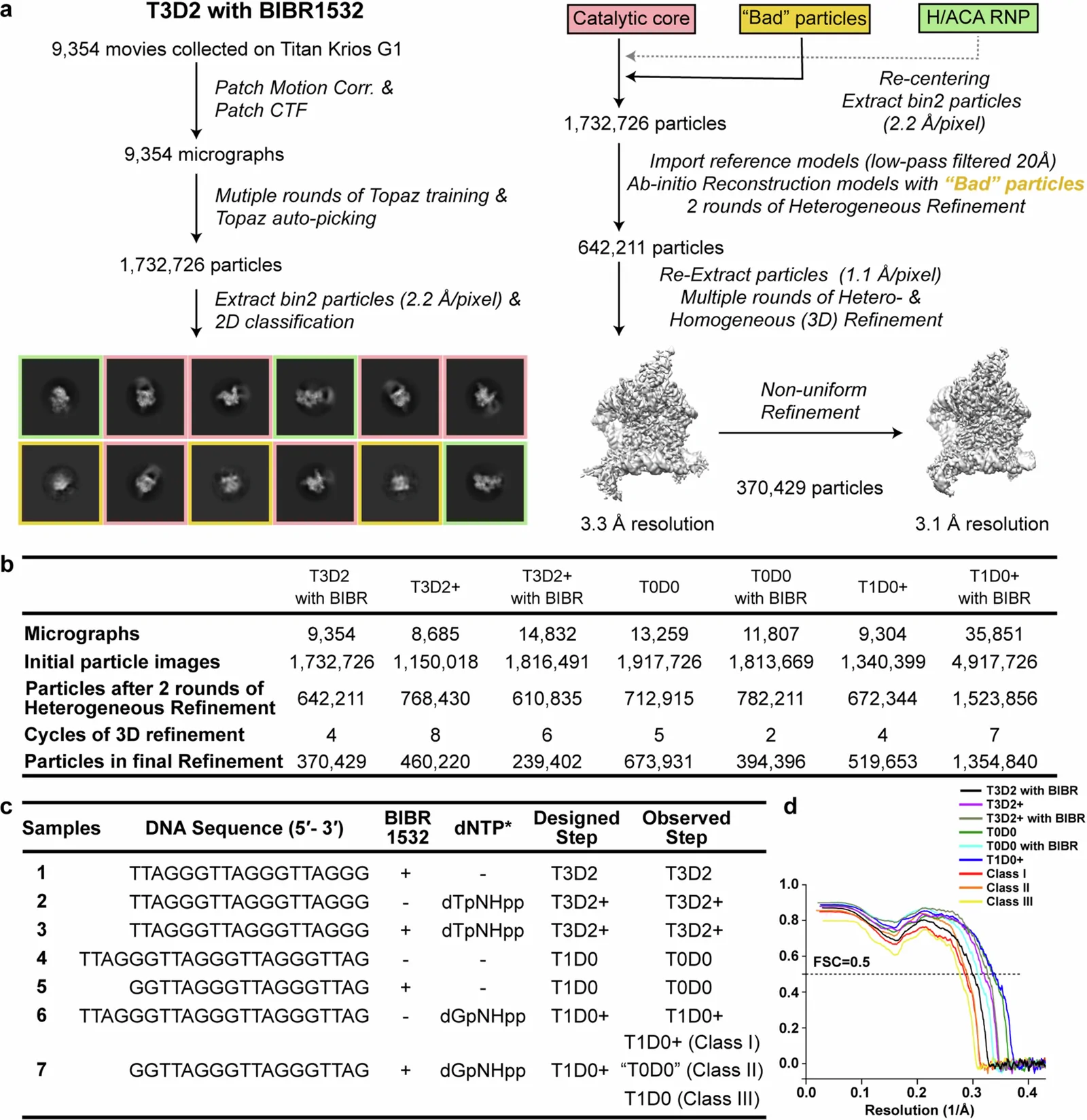
Cryo-EM data processing workflow of human telomerase–TPP1 with DNA primers and without or with BIBR1532 and/or dNTpNHpp. **a**, Summary of cryo-EM data processing in CryoSPARC showing workflow for the telomerase-TPP1 at T3D2 step with BIBR1532 as an example. **b**, Table showing relevant numbers of micrographs and particles for all datasets. **c**, Table showing components added to each sample. **d**, Fourier shell correlation (FSC) curves between atomic models and the corresponding cryo-EM maps. Class I, II, III refer to the three distinct classes resolved in **c** from the sample telomerase T1D0+ with BIBR1532.

**Extended Data Fig. 2:**
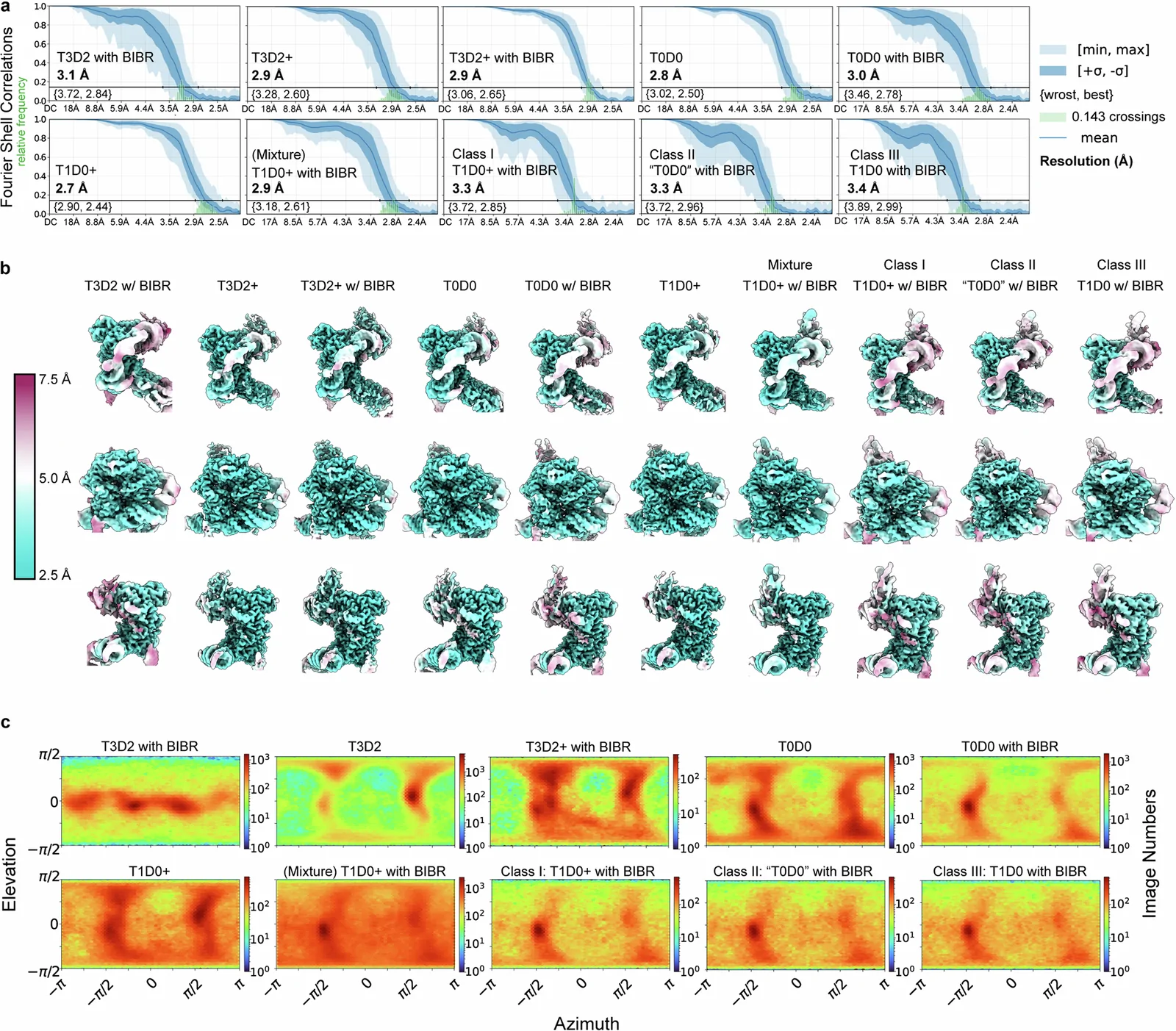
Evaluation of cryo-EM reconstructions. **a**, Plot of the Fourier shell correlation (FSC) both in Histogram and Directional as a function of the spatial frequency of the final reconstructions. The resolutions reported are based on the gold standard FSC with threshold value of 0.143. **b**, Local resolution evaluation of the reconstructions. **c**, Euler angle distribution of reconstructions.

**Extended Data Fig. 3:**
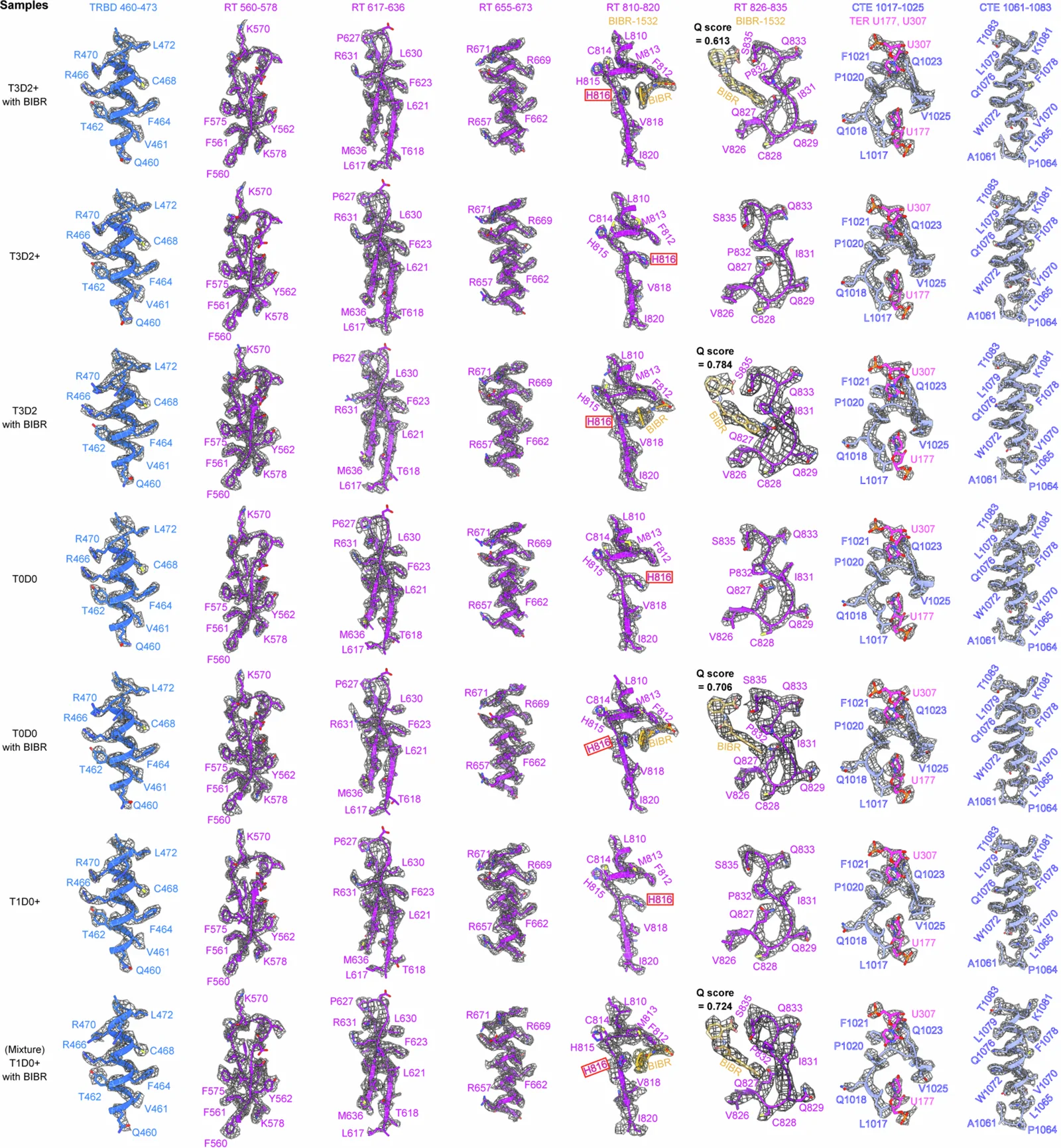
Evaluation of structure models. Representative cryo-EM densities encasing the corresponding atomic models of TERT RBD, RT and CTE regions for the telomerase samples at different steps of telomere repeat synthesis with or without BIBR1532. Densities and models at different steps in the absence or present of BIBR1532 are highly similar with the main differences being changes in sidechains in the BIBR binding pocket on RT domain, especially H816. Putative BIBR1532 binding region in CTE based on *Tc*TERT^16^ shows no difference with or without BIBR in the sample. Q score are calculated for ligand BIBR1532.

For cryo-EM studies, human telomerase in complex with DNA primer d(TTAGGG)_3_ and the shelterin protein TPP1 OB domain (hereafter referred to as TPP1) was purified as described^9^ and incubated with BIBR1532 before vitrification on cryo-EM grids (Extended Data Fig. 1). TPP1, which recruits telomerase to telomeres^24^, and a primer ending in dTTAGGG, which binds at the second step of telomere repeat addition (T3D2, where T3 is the template position in the active site and D2 is the DNA position relative to the template) (Fig. 1d), were used to allow direct comparison to previously reported human telomerase structures^6–9^. The overall structure was the same as in those studies (Fig. 1a). We verified that BIBR1532 inhibits the purified human telomerase using direct telomerase activity assays, which showed an IC_50_ of ~67 nM (Fig. 1e). Focused refinement of the catalytic core RNP resulted in a 3.1-Å-resolution cryo-EM map (Fig. 1d and Extended Data Figs. 1–3). The human telomerase catalytic core consists of TERT and two essential regions of TER: the template pseudoknot (t/PK) and conserved regions 4 and 5 (CR4/5) (Fig. 1b,d). Within TERT, the telomerase RNA-binding domain (TRBD), reverse transcriptase domain (RT, palm and fingers) and CTE (thumb) assemble into a TERT ring, while the telomerase essential N-terminal (TEN) domain and telomerase RAP motif (TRAP)^25^ form a complex critical for telomerase telomere repeat addition processivity above the catalytic cavity^9,26^ (Fig. 1c,d). TPP1 binds and stabilizes TEN–TRAP and increases telomerase activity^9^, while the histone H2A–H2B dimer binds near the three-way junction of the CR4/5 (refs. ^6–9^) (Fig. 1a,d).

**Fig. 2:**
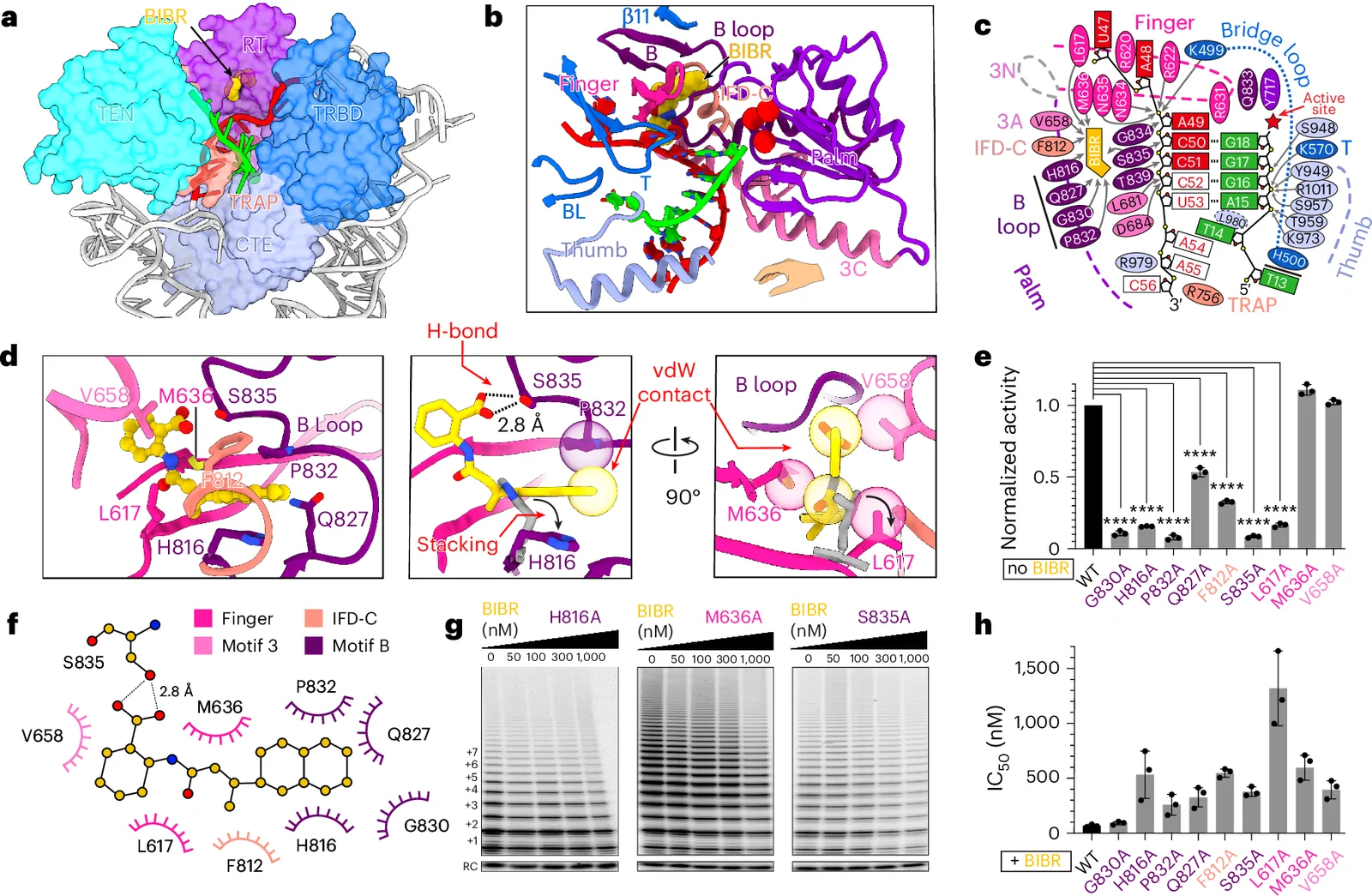
Structure of and interactions in the BIBR1532-binding pocket. **a**, Structure of telomerase catalytic core with DNA primer d(TTAGGG)_3_ and BIBR1532. TERT is shown as a semitransparent colored surface. TER is shown in gray except for the template (red). The DNA primer is shown in green. **b**, Ribbon representation highlighting the location of the BIBR1532-binding site relative to the active site, the template–DNA duplex and key TERT motifs involved in template–DNA duplex positioning. The three catalytic aspartates are shown as red spheres. The hand illustration provides orientation relative to other polymerases. **c**, Schematic showing TERT residue interactions with BIBR1532 and the template–DNA duplex. **d**, Left: ribbon model of the BIBR-binding pocket with key interacting residue side chains shown as sticks. BIBR1532 is shown in ball-and-stick representation. Middle and right: zoomed-in views showing conformational changes and interactions of residues with BIBR1532. The vdW interactions are shown as spheres. The side chains of H816 and L617 (gray) are from a structure without BIBR1532 (PDB 7TRE). **e**, Quantitation of direct telomerase activity assays for alanine substitutions within the BIBR-binding pocket in the absence of BIBR1532, relative to WT. Activity assays are shown in Extended Data Fig. 5. ****P < 0.0001, according to a one-tailed unpaired *t*-test. **f**, Two-dimensional plot of the interactions between BIBR1532 and TERT. **g**, Direct telomerase activity assays showing effects of BIBR1532 for individual alanine substitutions at H816, M636 and S832. Additional activity assays are in Extended Data Fig. 5. **h**, IC_50_ values calculated from direct telomerase activity assays measuring inhibition by BIBR1532 for individual alanine substitutions, plotted using GraphPad Prism (Methods). Data in **e** and **h** are presented as the mean ± s.d. from *n* = 3 biologically independent experiments. Colors in **a** and **b** are the same as in Fig. 1. Motifs in **c**–**h** are colored as indicated in **f**.

**Extended Data Fig. 4:**
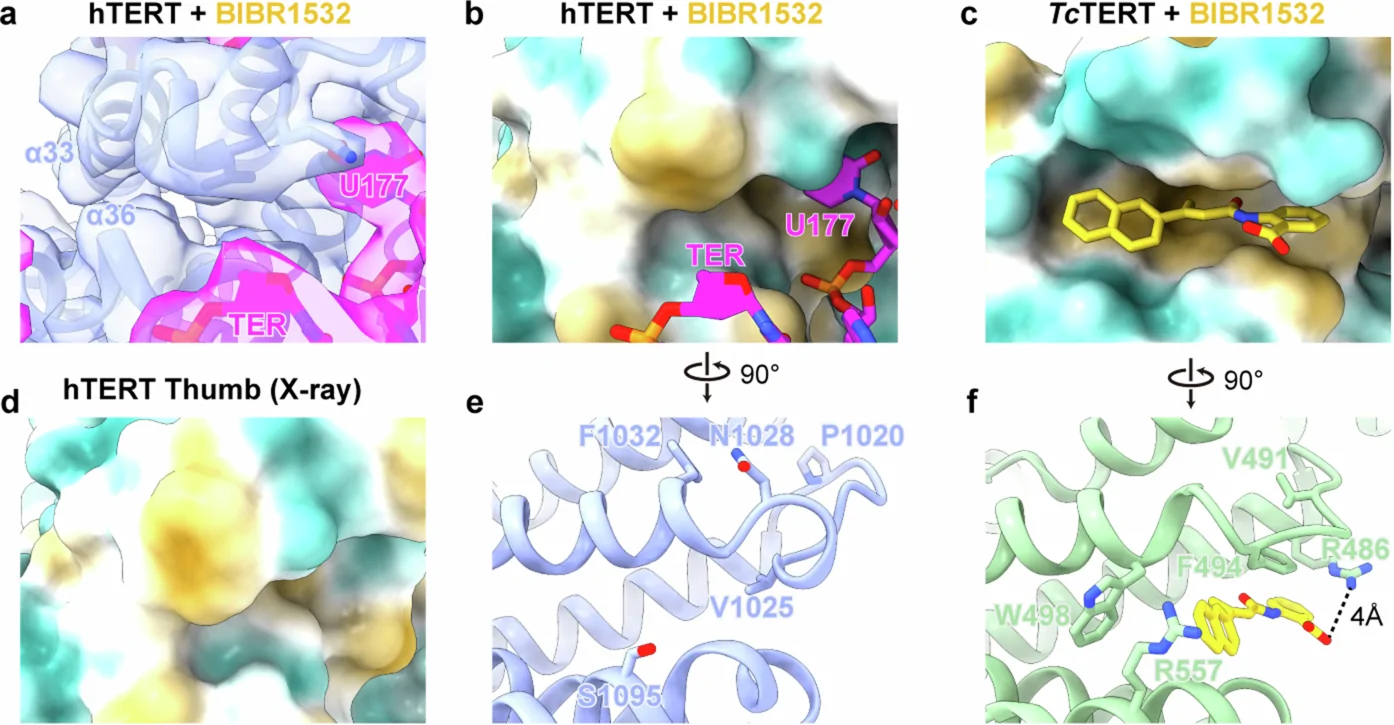
Comparison of BIBR binding pockets on human TERT and Tribolium casteneum TERT. **a**, Cryo-EM density and cartoon model of TERT CTE (thumb) region of human telomerase–TPP1 with BIBR1532 and d(TTAGGG)_3_ showing there is no density for BIBR1532. **b**, Surface view of panel **a**. **c**, Surface view of the structurally aligned (with hTERT) CTE region of *Tc*TERT with BIBR1532 bound (PDB ID: 5CQG). **d**, Surface view of the crystal structure of the CTE domain of human telomerase (PDB ID: 5UGW). **e**, Cartoon model of the CTE domain of human telomerase, with residues aligned by sequence to those in panel **f** (R486-P1020, V491-V1025, F494-N1028, W498-F1032, R557-S1095). **f**, Cartoon model of *Tc*TERT BIBR1532 binding pocket with BIBR, with key interacting residues shown as sticks.

Contrary to the expectation that BIBR1532 would bind human TERT CTE that was proposed to be homologous to the binding site observed in the crystal structure of the isolated primitive *Tc*TERT and disrupt TER–TERT interactions at helices α33–α36 (refs. ^9,16^), no cryo-EM density for BIBR1532 was observed in this region (Extended Data Figs. 3 and 4). Instead, we observed a distinct density within a deep pocket on the RT domain (Fig. 1d,f and Extended Data Fig. 3), whose size and shape in the cryo-EM map allowed accurate modeling of BIBR1532 (*Q* score = 0.613; Fig. 1f and Extended Data Fig. 3). This binding pocket (hereafter BIBR-binding pocket) is located on the RT domain (Figs. 1d,f and 2a), between the finger, which comprises a single β-hairpin formed by motifs 1–2, and the palm (Figs. 1c and 2b,c). The entrance of the pocket is partially covered by a flexible linker (residues 638–653, motif 3N) between motif 3A and the finger, the density of which is not visible for the most part apparently because of conformational dynamics (Figs. 1d and 2c). This linker may both limit the accessibility of the BIBR-binding pocket and conversely help maintain BIBR1532 in the binding pocket.

BIBR1532 adopts an obtuse angle conformation (Figs. 1f and 2c), with its small lipophilic head (Fig.1c) pointing outward from the BIBR-binding pocket and toward the template–DNA duplex (Figs. 1f and 2a–c). BIBR1532 does not directly interact with either the RNA template or the active-site residues but is in close proximity to both, ~6 Å and 12 Å away, respectively (Figs. 1f and 2a–c). BIBR1532 binding does not alter the structural features of the telomerase catalytic cavity previously reported, including the 4-bp template–DNA primer duplex^6,8,9,26^ (Figs. 1d and Fig. 2c). The binding pocket region differs only slightly from our previously published BIBR-free T3D2 structure^9^ (root-mean-square deviation (r.m.s.d.) = 1.22 Å; Extended Data Table 1). All key interactions that directly stabilize the template–DNA primer duplex remain unaltered by BIBR1532 binding, including those between the template and RT finger and motif B, contacts between the thumb loop and thumb helix in CTE, the TRBD bridge loop (BL) and the TRBD T-motif with the DNA primer^6,9^ (Fig. 2b,c). The structure is consistent with prior kinetic data showing that BIBR1532 is a direct, noncompetitive inhibitor of human telomerase^27^ that unprecedently binds within the RT domain without disrupting template–DNA interactions.

### Features of the BIBR-binding pocket

**Extended Data Fig. 5:**
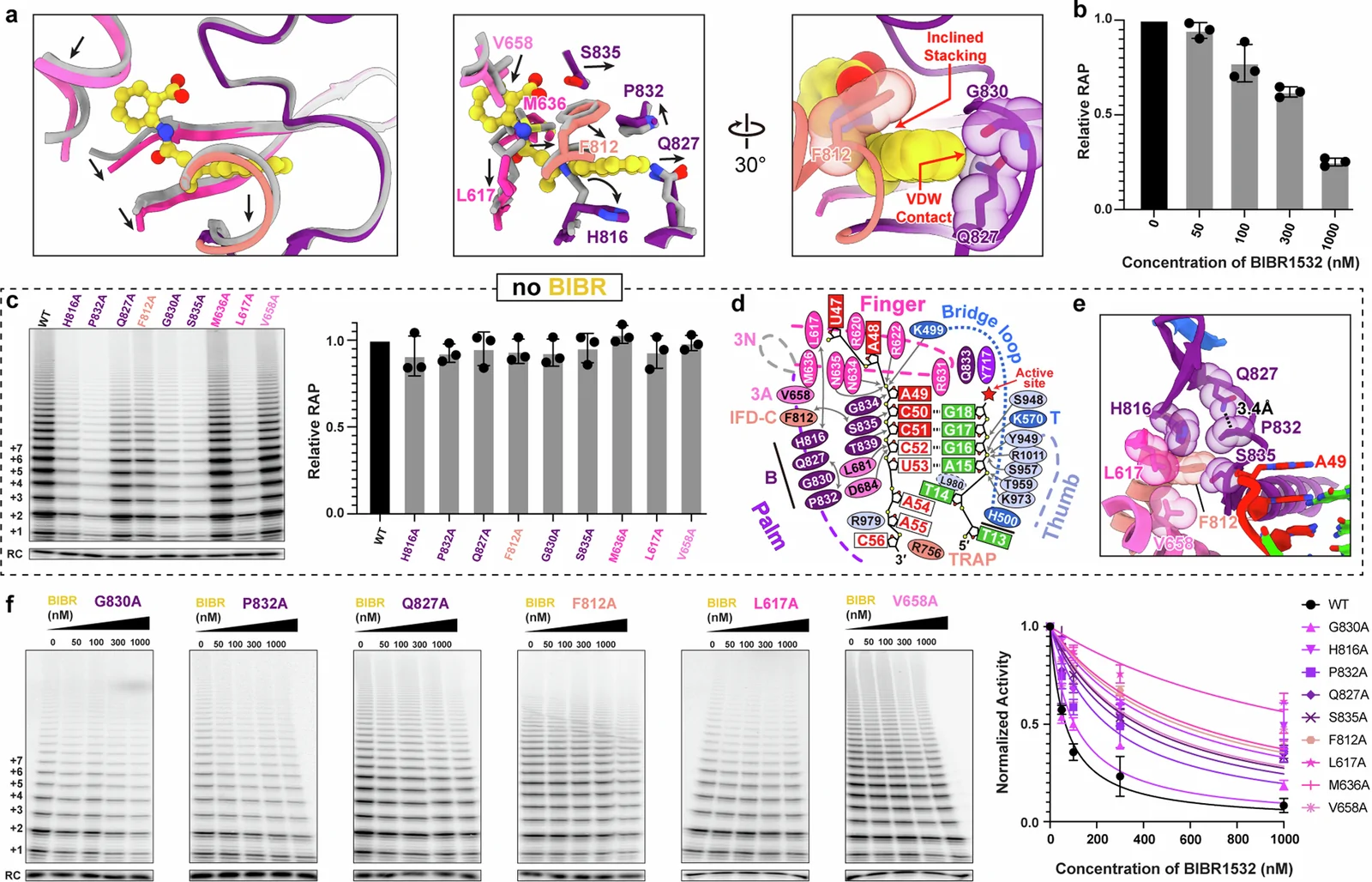
Structural and biochemical insights into BIBR binding pocket. **a**, Effect of BIBR1532 on TERT structure: (left) Superimposed ribbon models showing the movement of the backbone with (colored) and without (gray) BIBR1532 binding. (middle) Stick model showing the movement of side chains of BIBR binding pocket residues with (colored) and without (gray) BIBR1532 binding. (right) Zoomed-in view highlighting interactions between residues F812, G830, Q827, and BIBR1532. **b**, Dose-dependent effect of BIBR1532 on relative repeat addition processivity (RAP), normalized to TERT WT without BIBR1532. **c**, (left) Direct telomerase activity assays for individual alanine substitutions in the BIBR binding pocket. (right) Relative RAP plot of the assays. **d**, Schematic showing the interactions of BIBR binding pocket residues with each other and the template–DNA duplex in the absence of BIBR1532 binding. **e**, Ribbon and stick model of the BIBR binding pocket without BIBR1532 binding, illustrating interactions between residues S835, P832, H816, L617, and F812. Van der Waals interactions are depicted as spheres in **a** and **d**. **f**, (left) Direct telomerase activity assays showing the effects of BIBR1532 on individual alanine substitutions at G830, P832, Q827, F812, L617, and V658. (right) Inhibitor curve plot of the assays, plotted by GraphPad Prism (see Methods). All gels are representatives from 3 independent experiments. Plotted values are mean ± s.d.

The BIBR1532-binding pocket is formed on one side by adjacent β-hairpins from the finger (β12 and β13) and motif B (β18 and β19), which together with a β-strand from TRBD (β11) form a five-stranded β-sheet (Fig. 2b). A long motif B loop following the motif B β-hairpin wraps around the other side of BIBR1532 and the end of the motif 3 helix abuts the small lipophilic head (Fig. 2b–d). The N terminus of the helix connecting TRAP to the rest of the RT (IFD-C) also contributes to the binding pocket for the large lipophilic head (Figs. 1f and 2b–d). Residues from the finger (L617 and M636), motif 3 (V658), motif B (H816), motif B loop (G830, Q827 and P832) and IFD-C (F812) form a van der Waals (vdW) surface that accommodates BIBR1532 (Fig. 2d,f and Extended Data Fig. 5a). Motif B loop S835, the only residue in the BIBR-binding pocket that has a direct interaction with the template backbone, hydrogen bonds to the BIBR1532 carboxyl group (Fig. 2c,d,f). Upon BIBR1532 binding, although the backbone structure of TERT remains largely unchanged (Extended Data Table 1), H816 in motif B flips to stack with the BIBR1532 naphthalene moiety (Fig. 2d, center) and the L617 side chain in the finger shifts approximately 30° to accommodate BIBR1532 (Fig. 2d, right).

Individual alanine substitutions of residues forming the BIBR-binding pocket substantially decreased telomerase activity, except V658 and M636, but did not decrease RAP (Fig. 2e and Extended Data Fig. 5c). These results indicate that most of the residues that form the BIBR-binding pocket are important for activity despite all but S835 having no direct interactions with the template or DNA primer (Fig. 2c). Structural analysis of the region encompassing the BIBR-binding pocket (without BIBR1532), finger and template^9^ reveals an interaction network (Extended Data Fig. 5d,e). In the motif B loop, P832 interacts with Q827 and connects to residue Q833, which is inside the nucleotide-binding pocket. S835, which contacts the template nucleotide (C50) just below the nucleotide opposite the active site (A49), also forms vdW interactions with F812 from IFD-C. H816 in motif B exhibits a vdW contact with finger L617 (Extended Data Fig. 5e). Notably, H816D is a disease-associated substitution for pulmonary fibrosis^28^. Conversely, alanine substitutions of motif 3 V658 and finger M636 that do not affect telomerase activity are not part of this interaction network and M636 is dynamically positioned, according to the low-resolution cryo-EM density for its side chain (Extended Data Figs. 3 and 5d,e).

**Extended Data Fig. 6:**
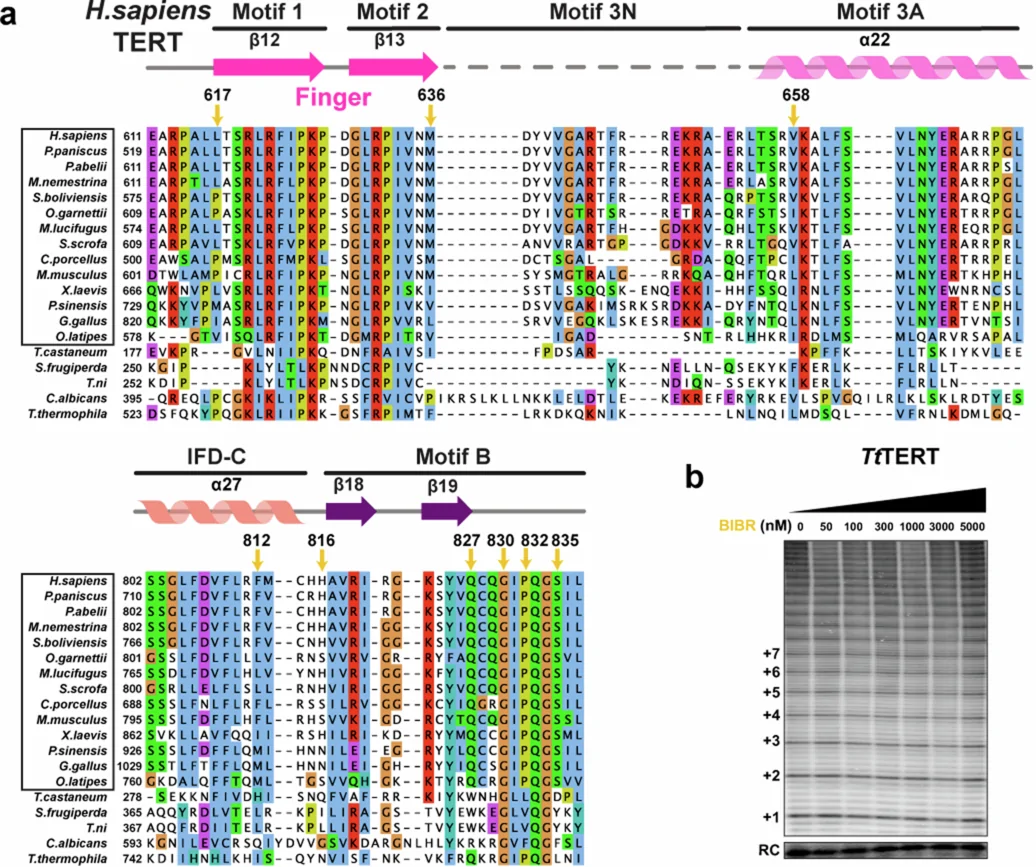
Sequence specificity of BIBR binding pocket. **a**, Multiple sequence alignment of the human TERT BIBR binding pocket region from vertebrates (boxed) and invertebrates. TERT secondary structure elements are indicated above the sequence. Numbers with yellow arrow indicate residues in the human telomerase BIBR binding pocket. Species abbreviations - *H.sapiens*: Human (hominoids). *P.paniscus*: Pygmy chimpanzee (hominoids). *P.abelii*: Sumatran orangutan (hominoids). *M.nemestrina*: Pig-tailed macaque (old world monkeys), *S.boliviensis*: Bolivian squirrel monkey (new world monkeys), *O.garnettii*: Small-eared galago (prosimian), *M.lucifugus*: Little brown bat (chiropteran), *S.scrofa*: Pig (artiodactyl), *C.porcellus*: Guinea pig (rodent), *M.musculus*: Mouse (rodent), *X.laevis*: African clawed frog (amphibian), *P.sinensis*: Chinese softshell turtle (reptile), *G.gallus*: Chicken (bird), *O.latipes*: Japanese rice fish (ray-finned fish), *T.castaneum*: Red flour beetle (anthropod), *S.frugiperda*: Fall armyworm (anthropod), *T.ni*: Cabbage looper (anthropod), *C.albicans*: Yeast (Fungus), *T.thermophila*: Ciliated (protozoan). **b**, Direct telomerase activity assay showing that BIBR1532 does not inhibit endogenously expressed, purified *Tetrahymena* telomerase. Three biologically independent experiments were performed, and a representative result is shown.

Having established the effect of alanine substitutions of residues in the BIBR-binding pocket in the absence of BIBR1532, we next determined their relative effect on telomerase activity in the presence of BIBR1532 (Fig. 2g,h, Extended Data Fig. 5f and Methods). BIBR1532 showed a dose-dependent decrease in wild-type (WT) telomerase activity and RAP (Fig. 1e and Extended Data Fig. 5b). A decrease in inhibition effect, as reflected in increased IC_50_ values (259 to 1,320 nM versus 67 nM for WT), was observed for alanine substitutions of all residues in the binding pocket except G830A, whose side chain points out of the binding pocket (Fig. 2g,h and Extended Data Fig. 5a,f), verifying their importance for BIBR1532 binding. M636A and V658A, which do not affect activity in the absence of BIBR1532, increased IC_50_ ~8-fold and 6-fold, respectively (Fig. 2d,f–h). L617 showed the most dramatic reduction in inhibition, consistent with its rearrangement upon and stabilization of BIBR1532 binding. Most residues in the BIBR-binding pocket are not conserved in *Tetrahymena* or *Tc*TERT (Extended Data Fig. 6a), explaining why BIBR1532 does not inhibit *Tetrahymena* telomerase activity (Extended Data Fig. 6b) and does not bind the RT domain in *Tc*TERT^16^. Notably, these pocket residues reside mostly within telomerase-specific motifs in the RT domain (Extended Data Fig. 6a), explaining why BIBR1532 is highly specific for telomerase versus other polymerases in human cells^12,29^.

Overall, these results show that the BIBR-binding pocket is within a structurally and functionally important region of the RT domain that is essential for telomerase catalysis and they indicate that disruption of the interaction network within the BIBR-binding pocket by alanine substitutions or BIBR1532 binding accounts for the observed effects on telomerase activity.

### Telomerase TERT has a unique rigid finger

**Extended Data Fig. 7:**
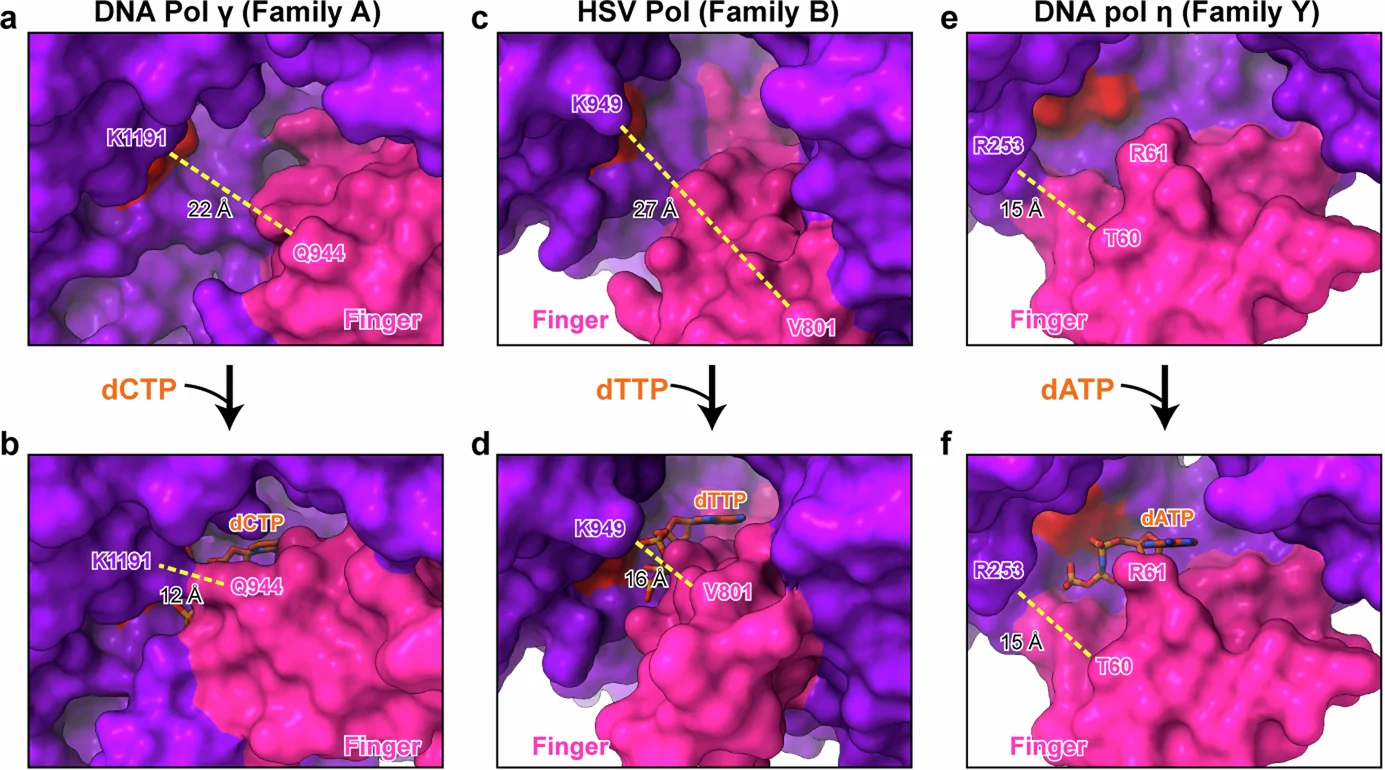
Finger movement (closure) upon dNTP binding in Family A (γ), Family B (HSV), and Family Y (η) DNA polymerases. **a**, Surface view of the ‘open’ active site in dNTP-free DNA polymerase γ (Family A DNA polymerase, PDB: 8UDL) and **b**, the ‘closed’ conformation upon dCTP binding (PDB:8D33). The shift of the finger domain (deep pink) is highlighted by the distance between the α-carbon atoms of K1191 and Q944. **c**, Surface view of the ‘open’ active site in dNTP-free HSV DNA polymerase (Family B DNA polymerase, PDB: 8OJ6) and **d**, the ‘closed’ conformation upon dTTP binding (PDB: 8OJ7). The shift of the finger domain (deep pink) is highlighted by the distance between the α-carbon atoms of K949 and V801. **e**, Surface view of the ‘static’ active site in dNTP-free DNA polymerase η (Family Y DNA polymerase, PDB: 3TQ1) and **f**, upon dTTP binding (PDB:3MR2). All dNTPs are shown in orange-red ball-and-stick. The static position of the finger domain (deep pink) is highlighted by the fixed distance between the α-carbon atoms of R253 and T60.

We next investigated whether BIBR1532 might affect conformational changes that occur during nucleotide addition, especially as two finger residues are part of the BIBR-binding pocket. During nucleotide addition, most DNA polymerases undergo a conformational change in the fingers domain, transitioning from an ‘open’ to a ‘closed’ state^30^. In the open conformation, the fingers domain is positioned away from the active site, allowing access for an incoming dNTP. Upon binding a correct dNTP complementary to the template nucleotide in the active site, the fingers domain closes toward the palm domain to facilitate positioning of the correct nucleotide for catalysis. This results in a large-scale movement of the fingers of up to 11 Å (refs. ^31–34^) (Extended Data Fig. 7).

**Fig. 3:**
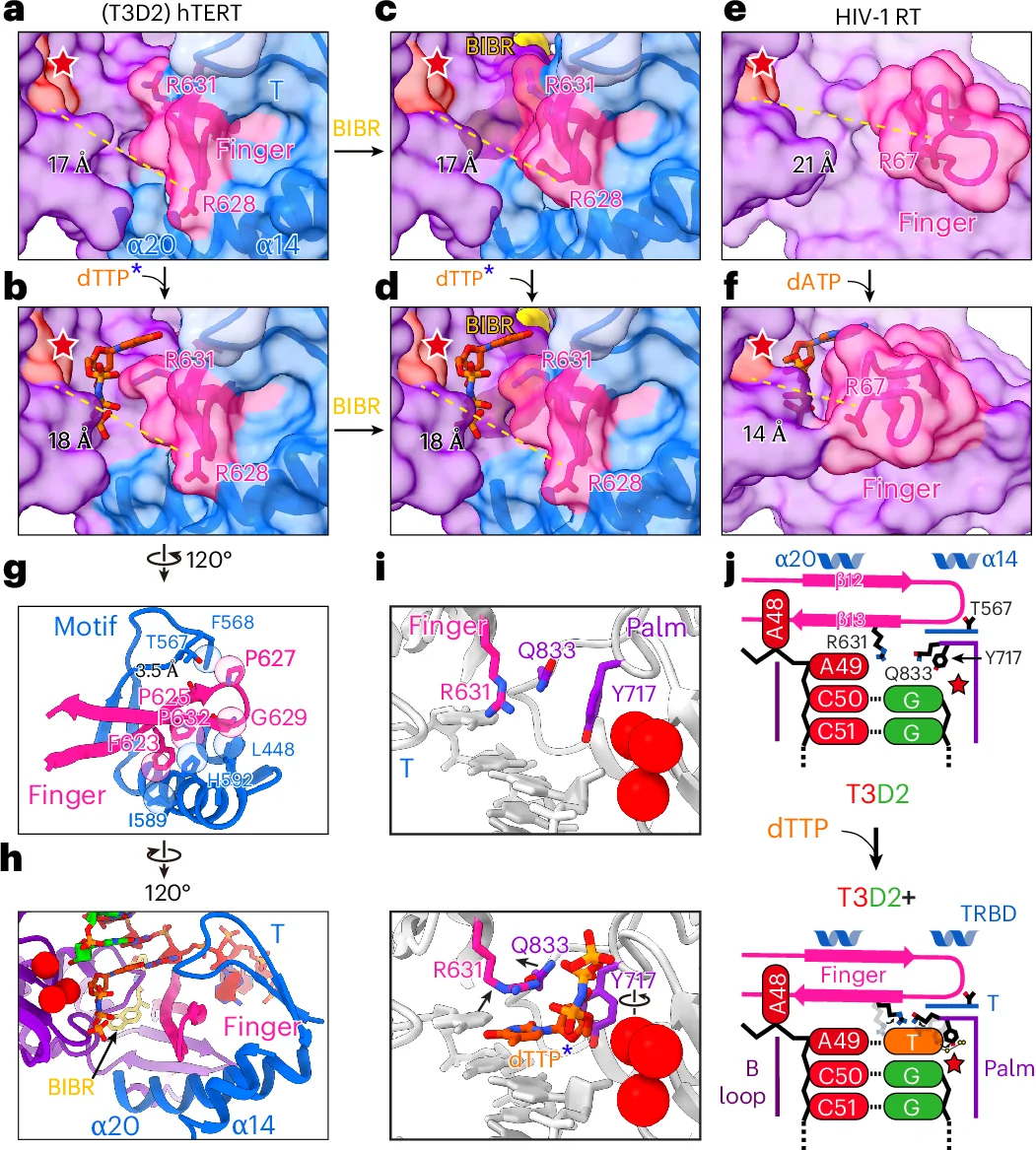
The finger domain of human TERT is clamped by the TRBD during catalysis, independent of BIBR1532 binding. **a**–**d**, Surface view of catalytic cavity of human telomerase: without dNTP (T3D2 step) (PDB 7TRE) (**a**), with dTpNHpp (indicated as dTTP*) (**b**), without dNTP and with BIBR1532 (**c**) and with dTpNHpp and BIBR1532 (**d**). **e**,**f**, Surface view of the catalytic cavity of HIV-1 RT without dNTP (PDB 8VB6; **e**) and with dATP (PDB 8VB7; **f**). The dNTPs are shown in orange-red ball-and-stick representation. Dashed yellow lines indicate the distance between the α-carbon of D110 and R67 for HIV-1 RT and of D712 and R628 for human TERT. **g**, Interactions between the TRBD and finger, with hydrogen bonds and vdW interactions indicated. **h**, View of secondary-structure elements around the active site, oriented the same as in **d**. **i**, Superimposed models of active-site region of human telomerase in dNTP-free state (top; PDB 7TRE) and with dTpNHpp bound (bottom), highlighting the side-chain movement of R631, Y717 and Q833. **j**, Schematics illustrating the nucleotide addition step from T3D2 to T3D2+.

To capture human telomerase bound to the telomeric DNA primer d(TTAGGG)_3_ and a nucleotide in the active site before catalysis, we introduced a nonhydrolyzable nucleotide analog of dTTP, 2′-deoxythymidine-5′-[(α, β)-imido]triphosphate (dTpNHpp), to form a ternary complex of telomerase–TPP1 with DNA primer and dTpNHpp (T3D2+, where + indicates dTpNHpp in the active site) (Extended Data Fig. 1c). Focused refinement of the catalytic core in cryo-EM datasets of telomerase–TPP1 at the T3D2+ step, without and with BIBR1532, yielded cryo-EM maps at 2.9-Å resolution (Extended Data Figs. 1–3). Unexpectedly, in contrast to what was expected on the basis of most DNA polymerases, all four structures (T3D2 (ref. ^9^), T3D2 with BIBR1532, T3D2+ and T3D2+ with BIBR1532) had remarkably similar active-site regions regardless of whether dTpNHpp and/or BIBR1532 was present (Fig. 3a–d and Extended Data Table 1). Notably, the finger loop in the nucleotide-binding pocket shifts <1 Å when dTpNHpp is added, with or without BIBR1532 (Fig. 3a–d). While human immunodeficiency virus (HIV-1) reverse transcriptase has a single β-hairpin finger like TERT, it has a 7-Å movement of its finger toward the active site (closed finger) (from 21 Å to 14 Å) when a dNTP binds^34^ (Fig. 3e,f). DNA polymerases generally have much larger fingers domains than HIV-1 RT and TERT^31–33^ (Extended Data Fig. 7).

Analysis of the structures reveals that, in human TERT, the finger movement is restricted by the TERT-specific TRBD (Fig. 3a–d,g,h). The TRBD T-motif and helices α14 and α20 together act as a molecular ‘clamp’ to immobilize the finger (Fig. 3a–d,g,h). The T-motif is a conserved TERT-specific sequence not found in other RTs^35^ that has a critical role in telomerase activity and processivity^36^. We identified several hydrophobic interactions between the finger and TRBD (T-motif F586–finger P627, α14 L448–finger I624, α20 590–finger F623 and α20 H592–finger P632) and a hydrogen bond between T-motif T567 and the finger P625 backbone (Fig. 3g). Notably, a T567 deletion has been identified as a disease mutation associated with pulmonary fibrosis^28^ and a T567M substitution reduces telomerase activity and RAP by ~50%^36^, consistent with functional importance of this interaction between the finger and T-motif. Together, these findings reveal that, unlike most other DNA polymerases including the closely evolutionarily related HIV-1 RT, TERT has a structurally constrained finger clamped by the TERT TRBD that prevents closure of the dNTP-binding pocket upon dNTP binding. Instead, the dNTP-binding pocket remains in a state that is about midway between the open and closed dNTP-binding pocket of HIV-1 RT regardless of dNTP and/or BIBR binding (Fig. 3a–f).

### Rigid finger explains telomerase high error and slow rate

Although no major conformational changes are observed in the active site on dNTP binding, side chains of several residues responsible for coordinating the incoming dNTP undergo structural rearrangements. In the binary T3D2 complex, three key residues, R631 (finger, motif 2), Y717 (steric gate, which excludes rNTPs^37^) and Q833 (palm, motif B′), occupy the nucleotide-binding pocket (Fig. 3i,j). When dTpNHpp binds the template to form the ternary complex (T3D2+), these residues shift to accommodate the incoming nucleotide. R631 and Q833 reposition above the nucleotide, forming a network of interactions that stabilize the phosphate moiety and correctly orient the nucleotide for catalysis (Fig. 3i,j), consistent with similar observations in *Tc*TERT^38^. Y717 rotates to lie near the deoxyribose C2′H, helping to sterically discriminate dNTPs from rNTPs that have a ribose C2′OH (Fig. 3i,j).

Except for the movement of these three side chains, the finger apparently remains relatively static throughout each step of nucleotide addition (Fig. 3i,j). In contrast, as discussed above, most DNA polymerases depend on dynamic finger movement between open and closed conformations to support efficient catalysis^39^ and high fidelity (error rate lower than 10^−4^ per nucleotide)^30^. Interestingly, Y-family DNA polymerases^40^ feature an intrinsically nondynamic finger that maintains an open, solvent-accessible binding pocket (Extended Data Fig. 7e,f), leading to slow catalysis^41^ and an inherently error-prone enzyme^42^. Similarly, telomerase shows both a slower catalytic rate^21^ and lower fidelity^43^ than canonical (A and B families) DNA polymerases, with an error rate of around 2 × 10^−3^ per nucleotide or roughly one noncanonical repeat per hundred canonical repeats^22^. The structural rigidity of the finger induced by interactions with the TRBD offers a structural explanation for these enzymological properties of telomerase.

### BIBR1532 disproportionately inhibits the first nucleotide addition

**Fig. 4:**
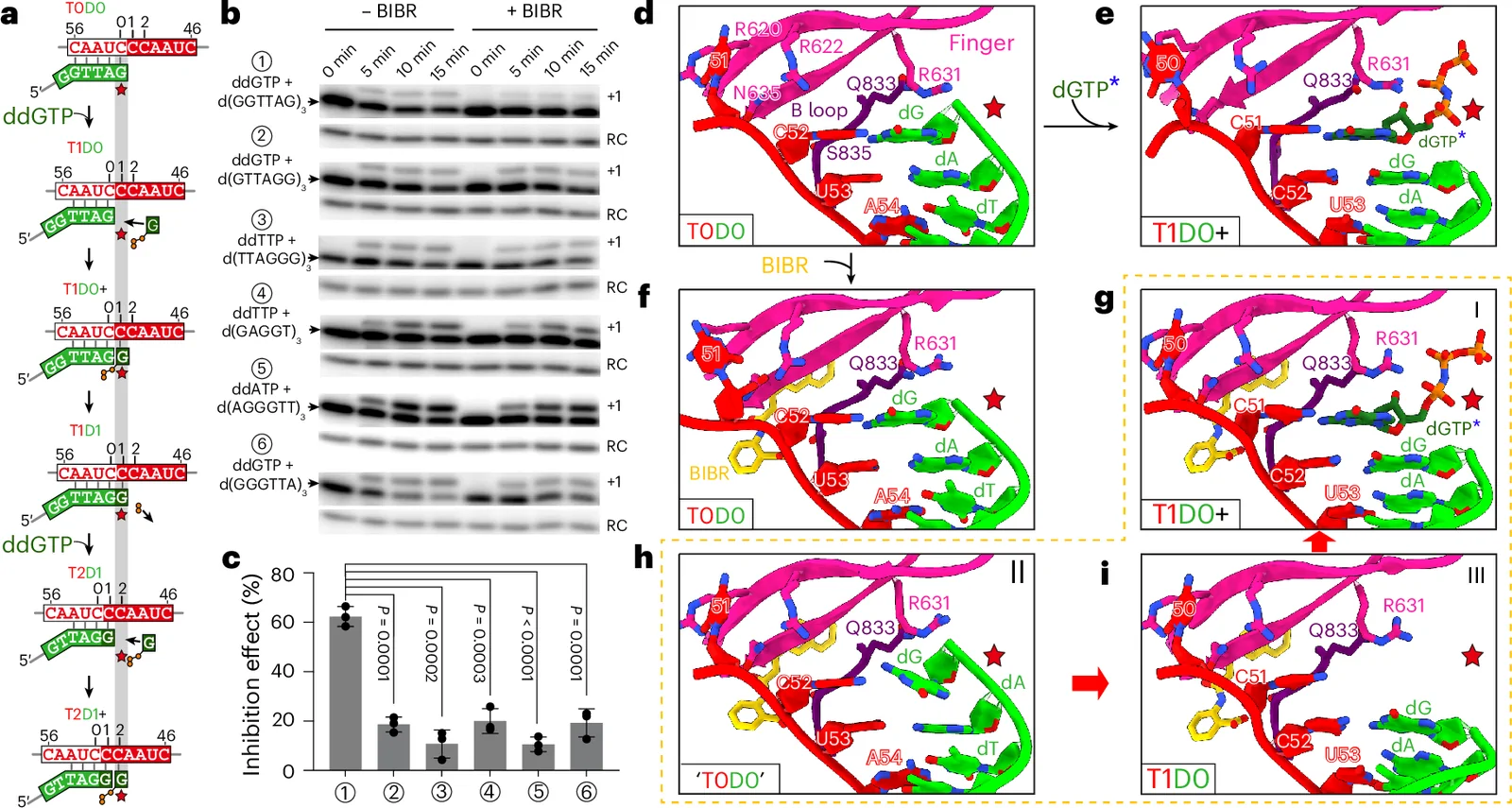
BIBR1532 disproportionally inhibits the slow first step of telomere repeat addition. **a**, Schematic design for the direct telomerase assay when using DNA primer d(GGTTAG)_3_ with ddGTP. The red star indicates the active site and ddGTPs are colored in forest green. **b**, Direct single-step telomerase assays without and with BIBR1532 for each step of the human telomerase nucleotide addition cycle, using 5′-end-labeled DNA primer and ddNTPs. Primers for each step are shown on the left. ‘+1’ indicates the products after incorporation of a single nucleotide. **c**, Quantification of BIBR1532’s inhibitory effect at each step of the human telomerase nucleotide addition cycle. Data are presented as the mean ± s.d. from *n* = 3 biologically independent experiments. *P* values were calculated using a one-tailed unpaired *t*-test. **d**–**i**, Ribbon-and-stick models of the active-site region for telomerase–TPP1 with DNA primer ending in d(GGTTAG): no BIBR or dGpNHpp (indicated as dGTP*), step T0D0 (**d**); with dGpNHpp, step T1D0+ (**e**); with BIBR1532, step T0D0 (**f**); with BIBR1532 and dGpNHpp, including class I, step T1D0+ (**g**), class II, step ‘T0D0’ (**h**) and class III, step T1D0 (**i**). The yellow dashed outline indicates three classes separated from one sample.

To investigate whether BIBR1532 has any selectivity for inhibition of individual steps of telomere repeat synthesis, we conducted a time-course single-step extension assay, targeting each of the six nucleotide addition steps. This assay used [^32^P]-end-labeled DNA primers and individual dideoxyribonucleoside triphosphates (ddNTPs), which lack a 3′-hydroxyl group and, thus, terminate DNA synthesis after a single nucleotide incorporation (Fig. 4a). Comparison of reactions with and without BIBR1532 revealed that BIBR1532 causes a modest reduction in activity (10–20%) across most steps but a much larger inhibitory effect (~60%) at the first step of nucleotide addition (Fig. 4b,c). This step has been identified as the rate-limiting first step in human telomere repeat synthesis, following rapid template translocation^44,45^. The observed dose-dependent reduction in RAP caused by BIBR1532 (Fig. 1e and Extended Data Fig. 5b) is consistent with its pronounced inhibitory effect on the rate-limiting initial step^23^.

**Extended Data Fig. 8:**
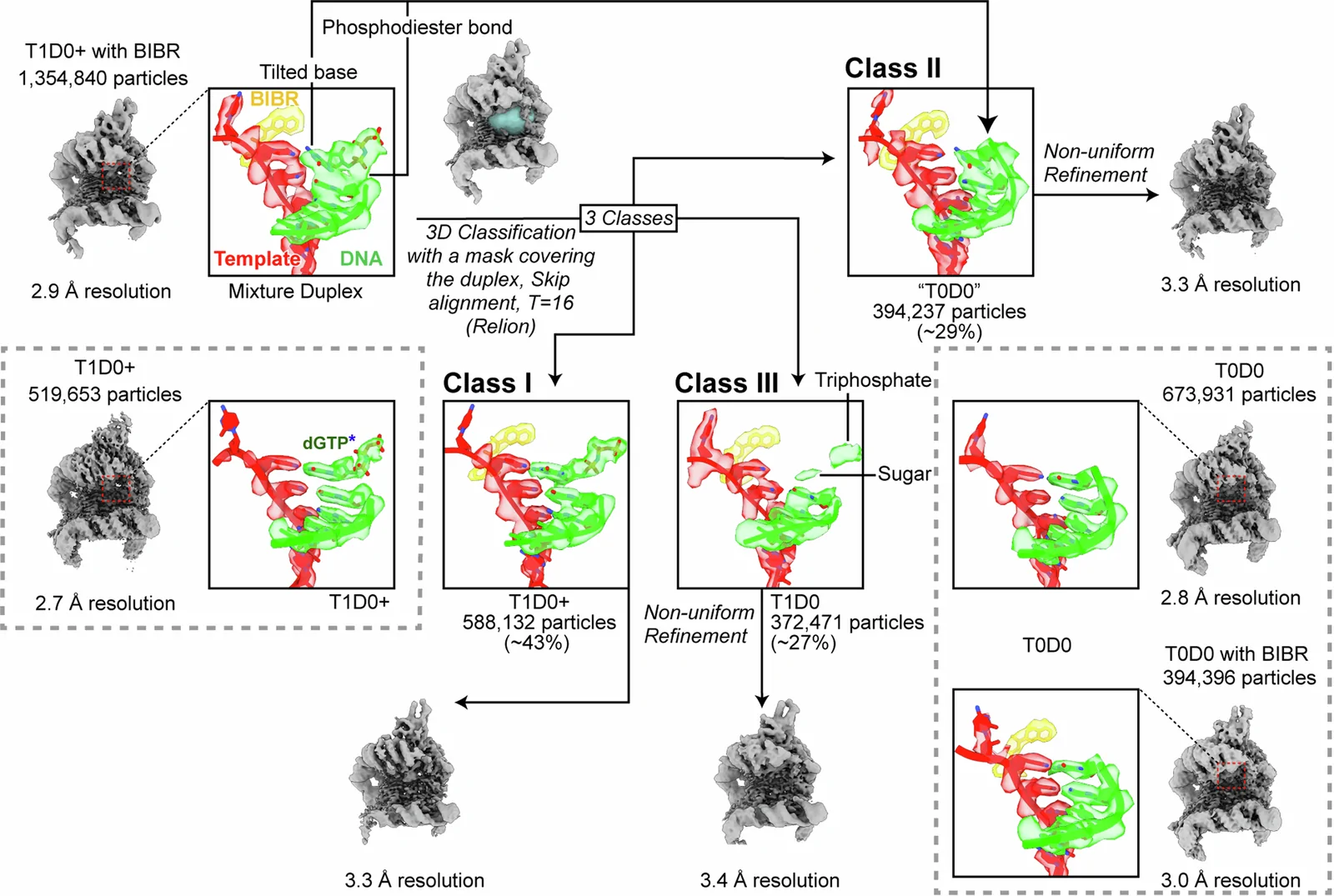
Focused 3D classification workflow of T1D0+ with BIBR1532. Reconstruction and focused 3D classification of human telomerase T1D0+ with BIBR1532. This map was further refined into three different classes, all with BIBR: I (T1D0 + ), II (T1D1), III (‘T0D0’) using the spherical mask shown in light blue. For comparison, the maps and models for template–DNA duplex regions from telomerase T1D0+ without BIBR, T0D0 without BIBR, and T0D0 with BIBR datasets are shown in gray dashed boxes.

To investigate the structural basis for the dramatic increase in inhibition by BIBR1532 at the first nucleotide addition, we collected cryo-EM data from human telomerase samples designed to trap the template alignment step and first step of telomere repeat synthesis. Telomerase–TPP1 was assembled with primers ending in d(GGTTAG) alone or with dGpNHpp and without or with BIBR1532 (Extended Data Fig. 1c). Focused refinement of the catalytic core RNP yielded cryo-EM maps at resolutions of 2.8 Å (T0D0), 3.0 Å (T0D0 with BIBR1532), 2.7 Å (T1D0+) and 2.9 Å (T1D0+ with BIBR1532) (Fig. 4d–i and Extended Data Figs. 1–3 and 8).

For telomerase–TPP1 with d(TTAGGG)_3_TTAG, instead of the four template–primer base pairs with template position 1 in the active site (T1D0) expected on the basis of previous structural studies at T3D2 step^6,8,9,26^, we observed a 5-bp duplex between the DNA primer and the alignment region of the template, with the last base pair at the active site (T0D0) (Fig. 4a,d and Extended Data Fig. 8). The same results (T0D0) were obtained for this sample with BIBR1532 added (Fig. 4f and Extended Data Fig. 8). Such occupancy of the telomerase active site was only previously observed with DNA primers containing three or four locked nucleic acid (LNA) nucleotides^26^. LNAs enhance duplex stability^46^, thereby apparently preventing duplex translocation out of the active site^26^, which is normally the fast step after nucleotide addition^47^.

In contrast to the results without a dNTP, when dGpNHpp was added to telomerase–TPP1 with d(TTAGGG)_3_TTAG, we observed the expected 4-bp RNA–DNA primer duplex and template position 1 and dGpNHpp in the active site (T1D0+) (Fig. 4e and Extended Data Fig. 8). For the dGTP analog to have entered the active site, the template–DNA duplex (T0D0) had to shift one step (nucleotide translocation) to T1D0+. These results explain why the first step of nucleotide addition is the slowest step; the initial duplex forms 5 bp, with the last bp positioned in the active site, and has to shift one step to the alignment region before the dGTP can enter the active site. It also explains why this step is dGTP concentration dependent^23,48^, as dGTP has to capture the occasional excursions of duplex out of active site. For all subsequent steps, the release of inorganic pyrophosphate (PPi) upon nucleotide addition helps drive the duplex nucleotide translocation, which is faster than nucleotide incorporation for telomerase^49^; we propose that this is because of the constitutively partially open nucleotide-binding pocket discussed above (Fig. 3a,i).

A strikingly different result was obtained when BIBR1532 was added to the sample with d(TTAGGG)_3_TTAG and dGpNHpp. In the initial modeling of the BIBR1532-bound cryo-EM map, we observed differences in the template–primer duplex region compared to the sample without BIBR1532 (Extended Data Fig. 8). Specifically, the cryo-EM density revealed a tilted and incompletely resolved base for the incoming dGpNHpp, along with disrupted duplex density, suggesting structural heterogeneity because of the presence of multiple conformational states (Extended Data Figs. 2 and 8). Subsequent three-dimensional (3D) classification revealed three distinct structural classes in the BIBR1532-bound dataset (Fig. 4g–i and Extended Data Fig. 8). The predominant class (class I, ~42%) corresponded to the expected T1D0+ state with the dGpNHpp paired in the active site and a 4-bp template–DNA primer helix (Fig. 4g and Extended Data Fig. 1c), as seen without BIBR1532. Class II (~29%) was an unexpected ‘T0D0’ intermediate state (Fig. 4h), in which the 3′ terminal dG of the primer occupies the active site in a tilted orientation, with only partial Watson–Crick pairing with template position 0 (C52), and dGpNHpp is not seen (Extended Data Fig. 8). Density for the rest of the 5-bp duplex is also weak, indicating conformational dynamics for this duplex. Class III apparently represents an intermediate T1D0 state (Fig. 4i), where partial density for the incoming dGpNHpp sugar and phosphate group was detected in the active site (Extended Data Fig. 8). The presence of these apparent intermediates (classes II and III) on the pathway to dGpNHpp occupying the active site at template position 1 (class I, T1D0+) indicates that BIBR inhibits the already slow extra nucleotide translocation step from T0D0 to T1D0/T1D0+ (Fig. 4a).

Overall, these activity assays and structures reveal a previously unknown rate-limiting substep for the first step of nucleotide addition (from T0D0 to T1D1/T2D1), where a stable 5-bp template–DNA duplex initially forms with the last bp in the active site (T0D0) and has to shift one base pair (T1D0) for the first dNTP to enter the active site (T1D0+) (Fig. 4a). The T0D0 to T1D0 substep is disproportionately affected by BIBR1532 binding.

### Mechanism of inhibition by BIBR1532

**Fig. 5:**
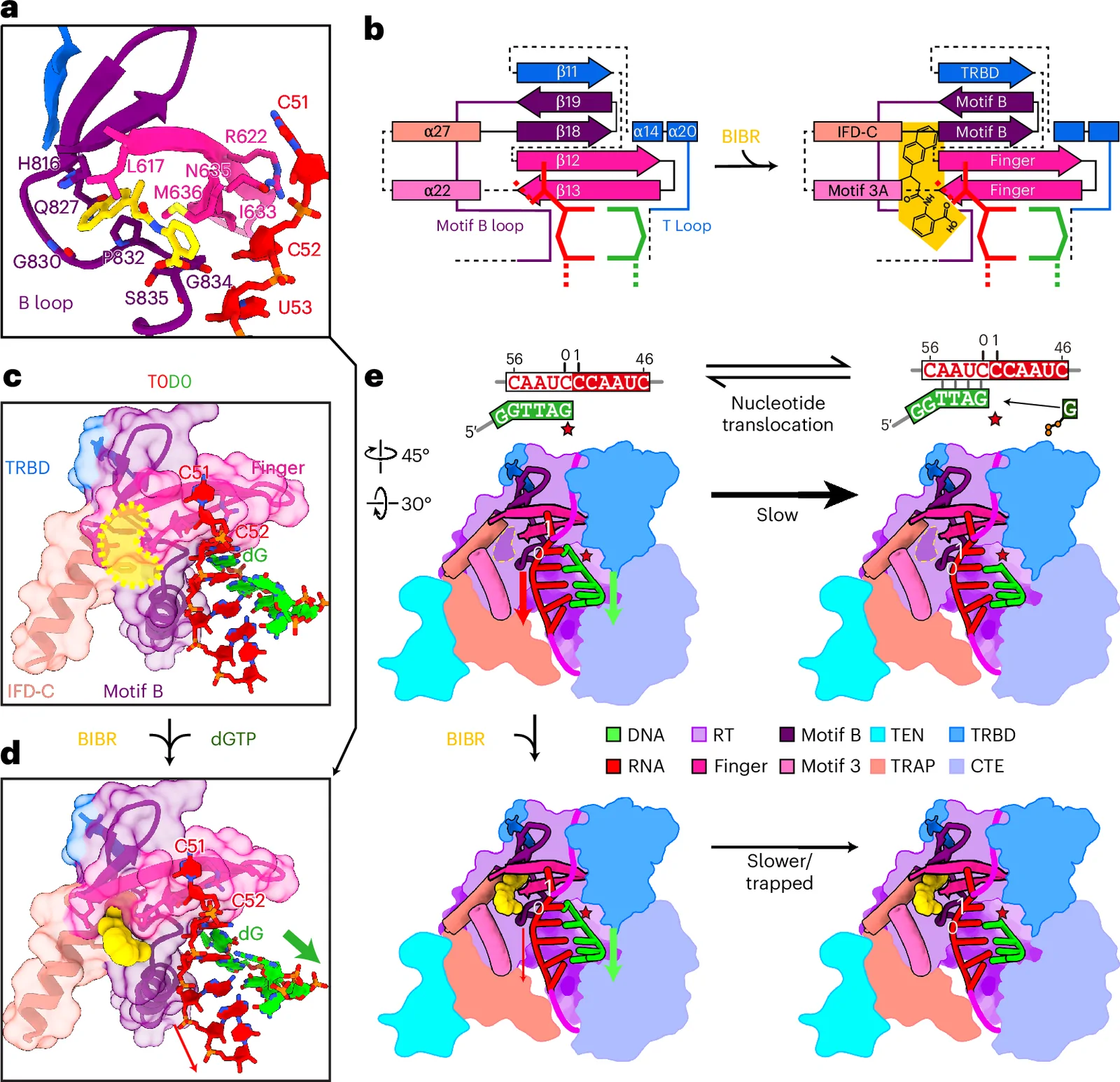
Mechanism of inhibition of telomerase nucleotide translocation by BIBR1532. **a**, Cartoon model illustrating the position of BIBR1532 relative to the structure elements involved in nucleotide translocation. **b**, Schematics of binding pocket without (left) and with (right) BIBR1532. **c**,**d**, Surface view of TERT region encompassing BIBR-binding pocket and template–DNA (T0D0 step) in the catalytic cavity without (**c**) and with (**d**) BIBR1532 and dGTP (dGpNHpp in cryo-EM sample). Arrows in **d** indicate the direction for asynchronous movement of DNA primer (green) followed by TER template (red) during nucleotide translocation. When BIBR1532 is bound, template movement is inhibited (indicated by thinner arrow). **e**, Schematic of mechanism of inhibition of human telomerase activity by BIBR1532. BIBR1532 inhibits the concerted dynamics of the TERT elements required for movement of the template during nucleotide translocation. The T0D0 to T1D0 step is illustrated.

On the basis of the location and structure of the BIBR-binding pocket, the effects of alanine substitutions within the binding pocket on telomerase activity and BIBR binding and the increased level of inhibition at the slow first step of telomere repeat synthesis, we propose a model for how BIBR1532 inhibits human telomerase (Fig. 5).

BIBR1532 binds in an allosteric pocket between the finger and palm motifs of the RT (Fig. 5a). It does not directly interfere with nucleotide addition because of the rigid finger domain that is clamped by the TRBD throughout the nucleotide addition cycle; rather, we propose that BIBR1532 restricts protein dynamics required for duplex repositioning after nucleotide addition (Fig. 5). Analysis of the BIBR-binding pocket, finger and template reveals that the five-stranded β-sheet and motif B loop form the same interaction network with the telomerase template in all of the structures (Fig. 5a–d). In the example shown for the T0D0 step, R622 and N635 in the finger (β12 and β13) interact with the phosphate group of template C52 (T0) and I633 stacks with the base of C52, separating it from C51 (T1), which is flipped away from the duplex (Fig. 5a). Individual alanine substitutions at the orthologous positions (R534A and I545A) (Extended Data Fig. 6a) in *Tetrahymena* TERT greatly reduce telomerase activity and RNA-binding affinity^50^. Within the motif B loop, G834 and S835 peptide backbone carbonyls form hydrogen bonds with nucleotides C52 and U53 ribose C2′OH, respectively (Fig. 5a). The same peptide residues successively interact with the new template nucleotides present at the same positions after each step of nucleotide translocation; that is, for T3D2, the interacting template nucleotides would be C50 (T2), A49 (T3) and A48 (T4) (Fig. 2c).

In the absence of BIBR1532, we propose that this whole functional unit (β-sheet and motif B loop) (Fig. 5b) undergoes coordinated motions that facilitate the stepwise movement of the template at each of the six nucleotide additions in telomere repeat synthesis. Previous molecular dynamics simulations suggested coordinated movement of this β-sheet with TRAP during catalysis^7^; however, those dynamics assume a closure of the finger domain similar to HIV-1 RT, which we find is not the case for TERT and is fundamentally different from what we propose here. Structural studies of RNA-dependent RNA polymerase and, more recently, Y-family DNA polymerase (Pol η) showed that, for nucleotide translocation, the product strand begins to move first, followed by the template^47,51^. Similarly, we proposed that, for *Tetrahymena* telomerase, the DNA primer would start to move, followed by the template^26^. Evidence for this in human telomerase is provided by the intermediate state ‘T0D0’ captured in telomerase with BIBR1532, d(GGTTAG)_3_ and dGpNHpp, where the first base pair is distorted and the duplex density is relatively weak (Fig. 4h and Extended Data Fig. 8). We note that this intermediate is only captured in the cryo-EM sample for the first step because BIBR1532 slows the template–primer movement in the already intrinsically slower first step. In the presence of BIBR1532, while the static TERT–template interactions remain unchanged (Fig. 5d,e), the inhibitor occupies the BIBR-binding pocket (Fig. 5c, yellow dotted line) within this functional unit, damping the dynamics required for the template to follow the DNA primer movement and consequently allosterically inhibiting the nucleotide translocation steps (Fig. 5b,d,e). This is particularly evident in the rate-limiting first step^23^, which is the only one where duplex translocation is not driven by the release of PPi (Fig. 4).

## Discussion

**Extended Data Fig. 9:**
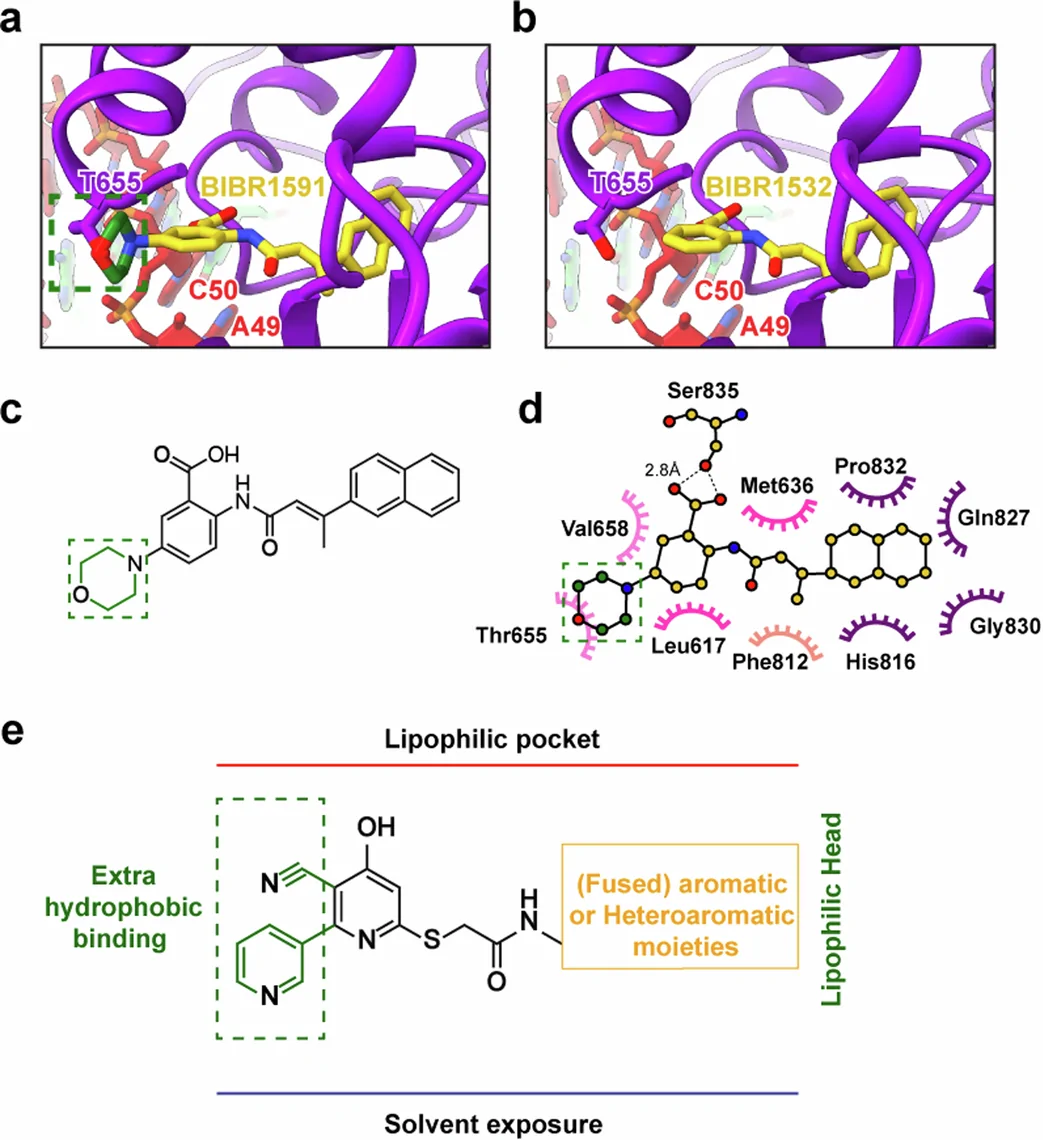
Structure-based development of BIBR derivatives. **a**, Model of BIBR binding pocket with BIBR1591 superimposed on the position of BIBR1532. The the morpholino head of BIBR1591 (green dashed box) would clash with T655 in Motif 3. BIBR1591 was superimposed with BIBR1532 using the align function in ChimeraX. **b**, A comparison view of BIBR1532. **c**, Chemical structure of BIBR1591 with its morpholino head in the green dashed box. **d**, Two-dimensional plot of the predicted interactions and clashes between BIBR1591 and TERT. **e**, Illustration of reported strategies for design of BIBR derivatives based on BIBR1532 binding pocket on CTE of *Tc*TERT^17–19^ that failed to improve inhibition.

Our study presents nine cryo-EM structures of human telomerase catalytic core RNP captured at two different steps of the telomere repeat addition cycle, without and with a dNTP analog in the active site and without and with the inhibitor BIBR1532. These structures reveal a novel binding pocket for BIBR1532 located between the finger and palm region of RT domain. Comparison of structures representing steps before and after catalysis, in the absence or presence of BIBR1532, revealed that telomerase has a unique rigid finger that helps explain its characteristic low activity and fidelity^21,43^. Unlike Y-family polymerases, which possess a structurally intrinsic stiff finger domain, the rigidity of telomerase’s finger is imposed by its interactions with motifs in the TRBD, a telomerase-specific domain important not only for binding TER^6,9^ but also for mechanism of telomerase. The results also demonstrate that the inhibition of telomerase by BIBR1532 is mechanistically different from inhibitors that target the movement of the finger, such as the small-molecule allosteric inhibitor of DNA polymerase θ^52^. The single-step direct telomerase assays and cryo-EM structures showed that BIBR1532 inhibits telomerase by affecting the movement of the template–DNA duplex needed to vacate the active site, especially during the first slow, rate-limiting step. These findings also explain why previous SAR studies to optimize the original BIBR1532 on the basis of the *Tc*TERT model have not produced more effective inhibitors. For example, BIBR1591, which was later proposed to form additional hydrophobic interactions in the *Tc*TERT binding pocket^17,18^, exhibits approximately fivefold weaker inhibition of human telomerase than BIBR1532 (ref. ^12^) (Extended Data Fig. 9). Instead, the modifications on BIBR1591 would point into the human TERT BIBR-binding pocket and sterically hinder its binding (Extended Data Fig. 9a–d). Our work also explains why modifications that enhance the hydrophobicity of the BIBR1532 headgroup did not improve inhibitory potency^17,18^ (Extended Data Fig. 9e). These structures of a telomerase inhibitor bound to telomerase explain the mechanism of telomerase inhibition by BIBR1532 and provide new insights into the catalytic cycle of telomerase, contributing to a further understanding of telomerase function and offering a rational pathway for SAR studies of BIBR derivatives.

## Methods

### Purification of human telomerase for cryo-EM sample preparation

Human telomerase was reconstituted in vivo and purified according to a previously reported method^9^. Briefly, human TERT and TER genes were separately cloned into pcDNA3.1 vectors with ZZ and Strep tag on TERT. HEK 293T cells, cultured on 150-mm plates with DMEM (Gibco) supplemented with 10% FBS (Atalanta), 1× GlutaMAX (Gibco) and 1× penicillin–streptomycin (Gibco), were used as the expression system for telomerase reconstitution. A 1:4 ratio of TERT and TER plasmids was used for cell transfection by calcium phosphate. After 48–72 h of transfection, the cells were collected by centrifugation at 1,000*g* for 5 min and resuspended in ice-cold HLB buffer (pH 8, 20 mM HEPES, 2 mM EDTA, 10% glycerol, 0.2 mM EGTA, 1 mM TCEP, 0.1% IGEPAL CA-630, 0.2% protease inhibitor cocktail, 0.2 mM phenylmethylsulfonyl fluoride and 10 μM MG-132). Cells were incubated on ice for 10 min before being flash-frozen and stored at −80 °C. Frozen cells from 150–180 plates were thawed and then subjected to two extra freeze–thaw cycles. The NaCl concentration of cell lysate was gradually adjusted to 300 mM and the solution was then incubated on ice for 30 min. Then, the NaCl concentration was diluted to 150 mM by adding an equal volume of HLB buffer to the cell extract. The insoluble cell debris was removed by ultracentrifugation (125,000*g*) for 1 h at 4 °C. The supernatant was incubated with mouse IgG−agarose (Sigma) for at least 5 h at 4 °C. The IgG−agarose resin was spun down and washed three times with wash buffer (pH 8, 20 mM HEPES, 150 mM NaCl, 2 mM EDTA, 0.2 mM EGTA, 1 mM TCEP, 10% glycerol and 0.1% IGEPAL CA-630) before incubation with tobacco etch virus protease at 4 °C for at least 1 h. The supernatant was subsequently incubated with streptavidin−agarose from *Streptomyces* *avidinii* (Sigma) prebound to 5′-biotinylated oligonucleotides at room temperature for at least 2.5 h. The resin was washed three times with wash buffer and eluted with 50 μM competitor oligonucleotide in 500 μl of wash buffer. The supernatant was further incubated with MagStrep XT3 resin (IBA LifeSciences) overnight at 4 °C. The beads were washed three times with wash buffer and incubated with 5 μM DNA primer d(TTAGGG)_3_, d(TTAGGG)_3_TTAG or d(GGTTAG)_3_ for 2–3 h (Extended Data Fig. 1c). DNA primers were all synthesized by Integrated DNA Technologies (IDT) DNA. The beads were finally washed three times with wash buffer and eluted with 20–35 μl of biotin elution buffer (pH 8, 20 mM HEPES, 150 mM NaCl, 2 mM MgCl2, 20 mM biotin, 1 mM TCEP and 0.05% IGEPAL CA-630) to obtain human telomerase. The TPP1 OB (residue 88–249) domain was purified and stored as previously described^9^ and 500 nM TPP1 OB was added to human telomerase and incubated for 1 h before vitrification.

### Cryo-EM sample preparation and data collection

The telomerase–TPP1 samples were variously prepared without or with addition of 1 µM BIBR1532 and without or with 1 mM dTpNHpp or dGpNHpp after purification and then incubated on ice for 30 min before grid preparation (Extended Data Fig. 1c). A 4-µl aliquot of the mixture was applied to Quantifoil gold grids coated with a GraFuture graphene oxide support film (Shuimu), which were glow-discharged at 15 mA for 10 s. The grids were then blotted at 10 °C using grade 595 filter paper (Ted Pella) and flash-frozen in a liquid propane–ethane mixture (63%:37%) with a Vitrobot Mark IV (Thermo Fisher) set to 100% humidity, blotting force set to 2 and blotting time of 2 s. These grids were subsequently examined with an FEI Tecnai TF20 transmission EM instrument to assess optimal ice thickness, particle concentration and particle distribution before data collection.

Cryo-EM data were collected on a Thermo Fisher Titan Krios G1 or G4 (Electron Imaging Center for Nanomachines (EICN) at University of California, Los Angeles (UCLA)) or G3 (S2C2 at Stanford Linear Accelerator Center (SLAC)) EM instruments, operated at 300 kV for automated data acquisition using SerialEM or EPU. Dose-fractionated video frames were captured with a Gatan K3 (G1 and G3) or Thermo Fisher Falcon 4i (G4) direct electron detector in super-resolution mode, achieving a pixel size of 0.55 Å at the sample level. A Gatan imaging filter (for G1 and G3) or Selectris X imaging filter (G4) was used with a slit width set to 20 eV. Before each imaging session, the microscope was precisely aligned and the parallel beam was optimized. The sample received a total dose rate of 40–50 e^−^ per Å^2^, divided into 40 frames. The total numbers of raw videos collected for different batches of samples are shown in Supplementary Table 1 and Extended Data Fig. 1.

### Cryo-EM data processing

The details of the cryo-EM data processing workflow are illustrated in Extended Data Fig. 1. All steps described below were performed with cryoSPARC^53^ unless otherwise indicated. All video stacks were subjected to patch motion correction (2× binned) and patch contrast transfer function (CTF) estimation. Particles were initially picked by blob picker and extracted with a box size of 400 pixels (1.1 Å per pixel) binned to 200 pixels (2.2 Å per pixel). These particles were subjected to two-dimensional (2D) classification and particles in good classes were selected to train a particle detection model for Topaz autopicking^54^. Particles picked by Topaz were cleaned by 2D classification, which generated high-quality classes with clear features of the catalytic core RNP and H/ACA RNP, as well as ‘bad’ classes of potential particles. These particles were subjected to two rounds of heterogeneous refinement using lowpass-filtered volumes of the catalytic core RNP (EMD-26086) and H/ACA RNP (EMD-26087) as initial models, together with some ‘bad’ initial models generated using particles from the ‘bad’ 2D classes through ab initio reconstruction. After screening, the particles from the classes of catalytic core RNP with good signal-to-noise ratio were reextracted with a box size of 400 pixels (1.1 Å per pixel) and several rounds of heterogeneous and homogeneous refinement were performed (Extended Data Fig. 1b). Candidate models and the particles within each class were selected and refined using nonuniform refinement and global CTF and local CTF refinement. Resolutions of the final cryo-EM maps were 2.7–3.1 Å (Extended Data Fig. 2 and Supplementary Table 1).

For sample T1D0+ with BIBR, 1,354,840 particles were finally picked from 35,851 micrographs (Extended Data Fig. 1b). To resolve the structural heterogeneity in the template–DNA duplex region, as previously described^26^, an additional round of focused 3D classification without alignment was performed using a spherical mask centered on this region (Extended Data Fig. 8). RELION (with alignment skipped and *T* value set to 16) could successfully separate the particles into three distinct classes with clear density: 588,132 particles corresponding to the T1D0+ step (class I), 394,237 particles corresponding to the ‘T0D0’ step (class II) and 372,471 particles corresponding to the T1D0 step (class III). Each class yielded reconstructions at resolutions of 3.3–3.4 Å (Extended Data Figs. 2 and 8 and Supplementary Table 1).

Map resolutions were estimated using the gold-standard Fourier shell correlation (FSC) at the 0.143 criterion^55^. Local resolution was assessed using the local resolution estimation tool in cryoSPARC. Data collection and processing statistics are provided in Supplementary Table 1.

### Model building and refinement

The atomic models of human telomerase catalytic core RNP bound to TPP1 and DNA primers, without or with BIBR1532 and without or with dTpNHpp or dGpNHpp were built and manually refined using Coot (version 0.9.8.92) as described previously^9^. The previously published structure of the human telomerase catalytic core with H2A/H2B (PDB 7TRF) served as the initial models. Real-space refinements of the final models were conducted using PHENIX (version 1.21)^56^ and ISOLDE^57^ and the completed models were validated in PHENIX (Extended Data Fig. 2 and Supplementary Table 1). Refinement statistics are summarized in Supplementary Table 1. All figures presenting models and maps, r.m.s.d. and *Q*-score calculation were prepared using UCSF Chimera^58^ and Chimerax^59^. The 2D plot of interactions between BIBR1532 and TERT was generated using LigPlot+^60^.

### Site-directed mutagenesis of human TERT

Individual alanine substitutions for residues in TERT (BIBR binding pocket) were generated using the Q5® site-directed mutagenesis kit (New England Biolabs) according to the standard protocol (https://www.neb.com/en-us/protocols/2013/01/26/q5-site-directed-mutagenesis-kit-protocol-e0554). In brief, primers were designed with NEBaseChanger and ordered from IDT DNA. PCR was performed to achieve exponential amplification, followed by a 5-min treatment with kinase, ligase and DpnI at room temperature. The resulting mixture was then transformed into DH5α competent *Escherichia* *coli* cells. Candidate colonies were selected and subjected to plasmid sequencing using Nanopore technology (Plasmidsaurus) to verify the site-specific mutagenesis.

### Direct telomerase activity assays with radiolabeled dGTP

Direct telomerase activity assays and purification of human and *Tetrahymena* telomerases were performed essentially as described^9,61^ but with BIBR1532 added at the start of reaction. For human telomerase, BIBR1532 concentration was optimized for exponential conditions (0–1,000 nM). Assays were conducted in a 20-μl reaction volume containing 25 mM Tris-HCl (pH 8.3), 2 mM MgCl_2_, 1 mM DTT, 500 μM dTTP, 500 μM dATP, 12.5 μM dGTP, 5 μCi of [α-^32^P]dGTP (PerkinElmer), 20 nM telomerase and 500 nM DNA primer d(TTAGGG)_3_. After incubating at 37 °C for 30 min, reactions were stopped by adding 80 μl quench buffer (10 mM Tris-HCl pH 8.0 and 10 mM EDTA) with a 15-nt P32 5′-end-labeled primer as a recovery control.

For *Tetrahymena* telomerase, assays were conducted in a 20-µl reaction volume containing 0–5,000 nM BIBR1532, 50 mM Tris-HCl (pH 8.0), 2 mM MgCl_2_, 1 mM spermidine, 2 mM TCEP, 200 µM dTTP, 3 µM dGTP, 5 µCi of [α-^32^P]dGTP (PerkinElmer), 20 nM telomerase and 1 µM telomeric d(GTTGGG)_3_ primer. The reactions were incubated at 30 °C for 30 min, after which they were terminated by adding 80 μl of quench buffer (10 mM Tris-HCl pH 8.0 and 10 mM EDTA) with a 15-nt ^32^P 5′-end-labeled primer as a recovery control.

Products were extracted with phenol–chloroform, ethanol-precipitated and resolved on a 10% denaturing polyacrylamide gel. The gel was vacuum-dried and exposed to a phosphor imaging (Cytiva) screen overnight. The final image was obtained by scanning the screen on a Typhoon biomolecular imager (Cytiva) and quantified with Image Lab (Bio-Rad). Telomerase activity was determined as previously described^9^. In brief, the integrated intensity of the lane that normalized to the recovery control was used to calculate telomerase activity. For activity assays with individual alanine substitution in TERT activity assay, activity was normalized to WT telomerase without BIBR1532.

For BIBR1532 inhibition activity assays, WT or mutant telomerase activity with BIBR1532 was normalized to WT or mutant telomerase activity without BIBR1532. Data were analyzed using GraphPad Prism 10 by nonlinear regression data fitting (inhibitor versus response) and IC_50_ values were obtained from the fitted plot for further comparison. For the single-turnover assays, activity was calculated as the +1 extension ratio, defined as the intensity of the +1 band divided by the intensity of the unextended primer band, with both values normalized to the recovery control in the same lane. Relative RAP plots are calculated using the fraction left behind method^45^.

### Direct telomerase activity assays with 5′-end-radiolabeled DNA primers and ddNTPs

Single-step direct telomerase activity assays were designed on the basis of previous studies^44,62^. PAGE-purified ^32^P 5′-end-labeled DNA primers, in combination with ddNTPs, were designed as follows: d(GGTTAG)_3_ with ddGTP for step 1, d(GTTAGG)_3_ with ddGTP for step 2, d(TTAGGG)_3_ with ddTTP for step 3, d(TAGGGT)_3_ with ddTTP for step 4, d(AGGGTT)_3_ with ddATP for step 5 and d(GGGTTA)_3_ with ddGTP for step 6 (Fig. 4b). The assays were conducted in a 20-µl reaction volume containing 25 mM Tris-HCl pH 8.3, 2 mM MgCl_2_, 1 mM DTT, 20 nM telomerase, 50 nM ^32^P 5′-end-labeled telomeric DNA primer and 250 µM ddNTPs, with or without 1 µM BIBR1532. After incubating at 37 °C for 0–15 min, the reactions were terminated by adding 80 µl of quenching buffer (10 mM Tris-HCl pH 8.0 and 10 mM EDTA) containing a 15-nt ^32^P 5′-end-labeled primer as a recovery control. Gel electrophoresis and imaging were performed as described above. The integrated intensity of the 18-nt primer band and the (19 + 1)-nt band was used to calculate telomerase activity at each step of the telomere synthesis cycle.

### Reporting summary

Further information on research design is available in the Nature Portfolio Reporting Summary linked to this article.

## Online content

Any methods, additional references, Nature Portfolio reporting summaries, source data, extended data, supplementary information, acknowledgements, peer review information; details of author contributions and competing interests; and statements of data and code availability are available at https://doi.org/10.1038/s41589-026-02238-6.

## Supplementary information

Supplementary Information Supplementary Table 1.

Reporting Summary

Peer Review File

## Source data

Source Data Figs. 1, 2 and 4 and Extended Data Figs. 5 and 6 Unprocessed source images for all electrophoretic gels.

## Figures and Tables

**Extended Data Table 1:**
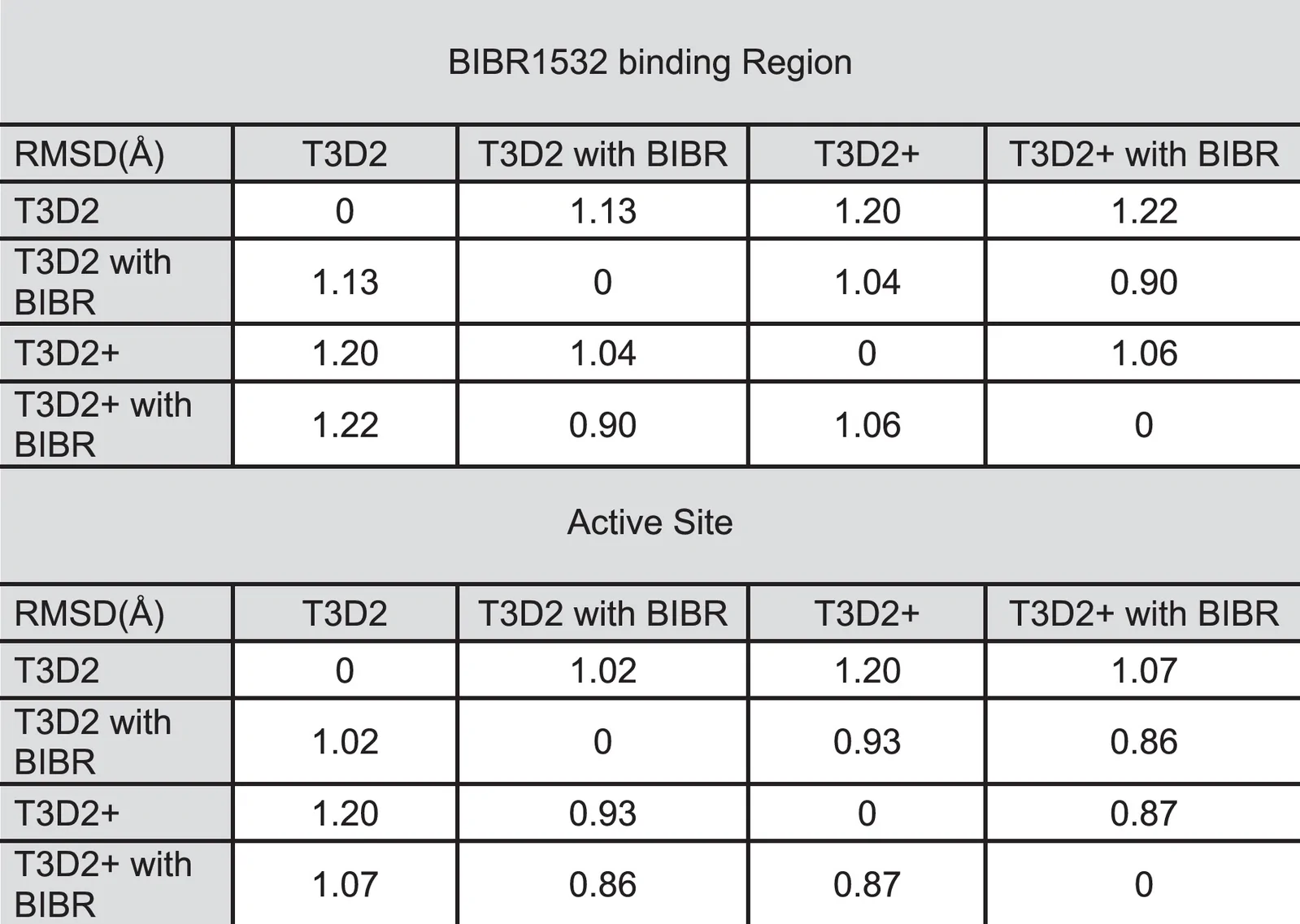
Root-mean-square deviation of regions of structures with and without BIBR1532

(Top)Root-mean-square deviation (RMSD) for the BIBR binding region shown in Fig. 5c and Fig. 5d (IFD-C, Motif B, finger, TRBD β-hairpin) and (Bottom) for the active site region shown in Fig. 3j (Motif A and B, finger, TRBD α14 and α20, T loop, DNA/RNA duplex) comparing the T3D2 and T3D2+ structures with and without BIBR1532.

## Data Availability

Cryo-EM density maps of the telomerase samples reported in this paper were deposited to the EM Data Bank under accession numbers EMD-72111 (T3D2 with BIBR1532), EMD-72112 (T3D2+), EMD-72113 (T3D2+ with BIBR1532), EMD-72114 (T0D0), EMD-72115 (T0D0 with BIBR1532), EMD-72117 (T1D0+), EMD-72118 (class I of T1D0+ with BIBR1532, T1D0+), EMD-72119 (class II of T1D0+ with BIBR1532, ‘T0D0’) and EMD-72120 (class III of T1D0+ with BIBR1532, T1D0). The atomic models were deposited in the Protein Data Bank under accession codes 9Q0Z (T3D2 with BIBR1532), 9Q10 (T3D2+), 9Q11 (T3D2+ with BIBR1532), 9Q12 (T0D0), 9Q14 (T0D0 with BIBR1532), 9Q16 (T1D0+), 9Q17 (class I of T1D0+ with BIBR1532, T1D0+), 9Q18 (class II of T1D0+ with BIBR1532, ‘T0D0’) and 9Q19 (class III of T1D0+ with BIBR1532, T1D0). Source data are provided with this paper.
