# Supplementary material for: Structures of human telomerase with BIBR1532 reveal novel mechanism of inhibition
Source: Nat Chem Biol. 2026 Jun 2;22(11):1854–62. doi: 10.1038/s41589-026-02238-6 (PMC13710158; doi:10.1038/s41589-026-02238-6)
Supplement: Supplementary file 1 [file 41589_2026_2238_MOESM1_ESM.pdf]

nature chemical biology

Supplementary information
https://doi.org/10.1038/s41589-026-02238-6

Structures of human telomerase with BIBR1532 reveal novel mechanism of inhibition

In the format provided by the authors and unedited

Supplementary Table 1: Cryo-EM data collection, refinement and validation statistics for human telomerase maps and model

	T3D2 with BIBR1532 (EMD-72111) (PDB 9Q0Z)	T3D2+ (EMD-72112) (PDB 9Q10)	T3D2+ with BIBR1532 (EMD-72113) (PDB 9Q11)	T0D0 (EMD-72114) (PDB 9Q12)	T0D0 with BIBR1532 (EMD-72115) (PDB 9Q14)
**Data collection and processing**					
Magnification	130,000	130,000	130,000	130,000	130,000
Voltage (kV)	300	300	300	300	300
Electron exposure (e–/Å^2)	45	49	43	46	50
Defocus range (μm)	−0.5 - −2.5	−0.5 - −2.5	−0.5 - −2.5	−0.5 - −2.5	−0.5 - −2.5
Pixel size (Å)	1.1	1.1	1.1	1.1	1.1
Symmetry imposed	C1	C1	C1	C1	C1
Final particle images (no.)	370,429	460,220	239,402	673,931	394,396
Map resolution (Å)	3.1	2.9	2.9	2.8	3.0
FSC threshold	0.143	0.143	0.143	0.143	0.143
Map resolution range (Å)	2.4 - 12.1	2.4 - 8.2	2.6 - 8.0	2.4 - 8.6	2.5 - 10.0
**Refinement**					
Initial model used (PDB code)	7TRF	7TRF	7TRF	7TRF	7TRF
CC (model vs. data)	0.73	0.77	0.82	0.79	0.60
Model resolution (Å)	3.4	3.1	3.0	3.0	3.5
FSC threshold	0.5	0.5	0.5	0.5	0.5
Map sharpening *B* factor (Å^2)	-105.3	-98.1	-83.7	-92.9	-96.0
Model composition					
Non-hydrogen atoms	14,707	14,836	14,931	14,578	14,747
Protein residues	1,252	1,266	1,275	1,258	1,275
RNA/DNA Nucleotides	223	223	223	215	215
Ligands	1	1	2	0	1
B factors (Å^2)					
Protein	45.14	60.16	59.89	58.94	165.32
Nucleotide	118.81	100.45	99.92	96.28	161.25
Ligand	20	20	20		20
R.m.s. deviations					
Bond lengths (Å)	0.004	0.004	0.003	0.003	0.003
Bond angles (°)	0.521	0.595	0.501	0.496	0.564
Validation					
MolProbity score	1.13	1.46	1.38	1.32	1.02
Clashscore	1.97	2.57	2.34	1.32	1.56
Poor rotamers (%)	1.12	0.18	0.18	0.28	0.09
Ramachandran plot					
Favored (%)	97.33	93.68	94.61	97.83	97.38
Allowed (%)	2.67	6.32	5.39	2.17	2.62
Disallowed (%)	0.00	0.00	0.00	0.00	0.00

	T1D0+ (EMD-72117) (PDB 9Q16)	Class I of T1D0+ with BIBR1532 (EMD-72118) (PDB 9Q17)	Class II of T1D0+ with BIBR1532 (EMD-72119) (PDB 9Q18)	Class III of T1D0+ with BIBR1532 (EMD-72120) (PDB 9Q19)
**Data collection and processing**				
Magnification	130,000	130,000	130,000	130,000
Voltage (kV)	300	300	300	300
Electron exposure (e–/Å^2)	48	50	50	50
Defocus range (μm)	−0.5 - −2.5	−0.5 - −2.5	−0.5 - −2.5	−0.5 - −2.5
Pixel size (Å)	1.1	1.1	1.1	1.1
Symmetry imposed	C1	C1	C1	C1
Final particle images (no.)	519,653	588,132	394,237	372,471
Map resolution (Å)	2.7	3.3	3.3	3.4
FSC threshold	0.143	0.143	0.143	0.143
Map resolution range (Å)	2.4 - 8.5	2.7 - 11.2	2.7 - 10.8	2.8 - 11.4
**Refinement**				
Initial model used (PDB code)	7TRF	7TRF	7TRF	7TRF
CC (model vs. data)	0.71	0.70	0.60	0.68
Model resolution (Å)	3.0	3.5	3.6	3.6
FSC threshold	0.5	0.5	0.5	0.5
Map sharpening *B* factor (Å^2)	-86.6	-97.6	-89.7	-81.3
Model composition				
Non-hydrogen atoms	14,560	14,585	14,747	14,554
Protein residues	1,250	1,250	1,275	1,250
RNA/DNA Nucleotides	216	216	215	216
Ligands	1	2	1	1
B factors (Å^2)				
Protein	43.61	43.61	165.32	43.61
Nucleotide	95.24	95.24	161.65	95.24
Ligand	20	20	20	20
R.m.s. deviations				
Bond lengths (Å)	0.003	0.003	0.004	0.003
Bond angles (°)	0.510	0.615	0.703	0.618
Validation				
MolProbity score	1.07	1.29	1.09	1.26
Clashscore	1.73	4.05	2.40	2.69
Poor rotamers (%)	1.02	0.09	0.00	0.00
Ramachandran plot				
Favored (%)	97.33	97.49	97.70	96.76
Allowed (%)	2.67	2.51	2.30	3.24
Disallowed (%)	0.00	0.00	0.00	0.00
