# Supplementary material for: Structures of human telomerase with BIBR1532 reveal novel mechanism of inhibition
Source: Nat Chem Biol. 2026 Jun 2;22(11):1854–62. doi: 10.1038/s41589-026-02238-6 (PMC13710158; doi:10.1038/s41589-026-02238-6)
Supplement: Supplementary file 2 [file 41589_2026_2238_MOESM2_ESM.pdf]

nature portfolio

Corresponding author(s): Juli Feigon

Last updated by author(s): Mar 25, 2026

Reporting Summary

Nature Portfolio wishes to improve the reproducibility of the work that we publish. This form provides structure for consistency and transparency in reporting. For further information on Nature Portfolio policies, see our Editorial Policies and the Editorial Policy Checklist.

Statistics

For all statistical analyses, confirm that the following items are present in the figure legend, table legend, main text, or Methods section.

n/a	Confirmed	
☐	☒	The exact sample size (*n*) for each experimental group/condition, given as a discrete number and unit of measurement
☐	☒	A statement on whether measurements were taken from distinct samples or whether the same sample was measured repeatedly
☐	☒	The statistical test(s) used AND whether they are one- or two-sided<br>*Only common tests should be described solely by name; describe more complex techniques in the Methods section.*
☒	☐	A description of all covariates tested
☒	☐	A description of any assumptions or corrections, such as tests of normality and adjustment for multiple comparisons
☐	☒	A full description of the statistical parameters including central tendency (e.g. means) or other basic estimates (e.g. regression coefficient) AND variation (e.g. standard deviation) or associated estimates of uncertainty (e.g. confidence intervals)
☐	☒	For null hypothesis testing, the test statistic (e.g. *F*, *t*, *r*) with confidence intervals, effect sizes, degrees of freedom and *P* value noted<br>*Give P values as exact values whenever suitable.*
☒	☐	For Bayesian analysis, information on the choice of priors and Markov chain Monte Carlo settings
☒	☐	For hierarchical and complex designs, identification of the appropriate level for tests and full reporting of outcomes
☒	☐	Estimates of effect sizes (e.g. Cohen's *d*, Pearson's *r*), indicating how they were calculated

Our web collection on statistics for biologists contains articles on many of the points above.

Software and code

Policy information about availability of computer code

Data collection	SerialEM 4.2, Smart EPU
Data analysis	CryoSPARC 4.0, RELION 4.0, Topaz 0.2.4, Coot 0.9.8.92, UCSF Chimera 1.15 and ChimeraX 1.1, PHENIX 1.21, Ramachandran (within PHENIX 1.21), PROMALS3D (web server), ESPript 3.0.8 (web server), LigPlot + v.2.3, Image Lab 6.1, GraphPad Prism 10

For manuscripts utilizing custom algorithms or software that are central to the research but not yet described in published literature, software must be made available to editors and reviewers. We strongly encourage code deposition in a community repository (e.g. GitHub). See the Nature Portfolio guidelines for submitting code & software for further information.

Data

Policy information about availability of data

All manuscripts must include a data availability statement. This statement should provide the following information, where applicable:

- Accession codes, unique identifiers, or web links for publicly available datasets
- A description of any restrictions on data availability
- For clinical datasets or third party data, please ensure that the statement adheres to our policy

Cryo-EM density maps of the telomerase samples reported in this manuscript have been deposited in the Electron Microscopy Data Bank under accession numbers EMD-72111 (T3D2 with BIBR1532), EMD-72112 (T3D2+), EMD-72113 (T3D2+ with BIBR1532), EMD-72114 (T0D0), EMD-72115 (T0D0 with BIBR1532), EMD-72117 (T1D0+), EMD-72118 (Class I of T1D0+ with BIBR1532, T1D0+), EMD-72119 (Class II of T1D0+ with BIBR1532, "T0D0"), and EMD-72120 (Class III of T1D0+ with

BIBR1532, T1D0). The atomic models have been deposited in the Protein Data Bank under accession codes 9Q0Z (T3D2 with BIBR1532), 9Q10 (T3D2+), 9Q11 (T3D2 + with BIBR1532), 9Q12 (T0D0), 9Q14 (T0D0 with BIBR1532), 9Q16 (T1D0+), 9Q17 (Class I of T1D0+ with BIBR1532, T1D0+), 9Q18 (Class II of T1D0+ with BIBR1532, "T0D0"), and 9Q19 (Class III of T1D0+ with BIBR1532, T1D0).

Research involving human participants, their data, or biological material

Policy information about studies with human participants or human data. See also policy information about sex, gender (identity/presentation), and sexual orientation and race, ethnicity and racism.

Reporting on sex and gender	No data on sex or gender information was collected for our studies.
Reporting on race, ethnicity, or other socially relevant groupings	No data on race, ethnicity, or other socially relevant groupings were collected in our studies.
Population characteristics	No data on population characteristics was collected for our studies.
Recruitment	No participant was recruited for our studies.
Ethics oversight	No ethics oversight was required or involved in our studies.

Note that full information on the approval of the study protocol must also be provided in the manuscript.

Field-specific reporting

Please select the one below that is the best fit for your research. If you are not sure, read the appropriate sections before making your selection.

☒ Life sciences ☐ Behavioural & social sciences ☐ Ecological, evolutionary & environmental sciences

For a reference copy of the document with all sections, see nature.com/documents/nr-reporting-summary-flat.pdf

Life sciences study design

All studies must disclose on these points even when the disclosure is negative.

Sample size	No statistical method was used to predetermine sample size. Cryo-EM 3D reconstructions were calculated from 103,092 images (14.6 million particles) from 7 separate imaging sessions. The data size is comparable to other studies in this field. The number of particles used for each final map is sufficient to obtain reliable classification and reconstruction results by cryo-EM.
Data exclusions	For cryo-EM analysis, particles that do not belong to the class of interest or have poor qualities based on well established cryoEM principle were excluded after rounds of 2D and 3D classification. This is standard practice required to obtain high resolution cryo EM structure of the class of interest. For functional studies, no data were excluded from any analysis.
Replication	All biochemical experiments were repeated three times and are all reproducible.
Randomization	No grouping required for our studies.
Blinding	Blinding was not relevant to this study, because the study does not involve human subjects or live animals and no grouping was conducted for telomerase activity assays.

Reporting for specific materials, systems and methods

We require information from authors about some types of materials, experimental systems and methods used in many studies. Here, indicate whether each material, system or method listed is relevant to your study. If you are not sure if a list item applies to your research, read the appropriate section before selecting a response.

Materials & experimental systems

n/a	Involved in the study	
☒	☐	Antibodies
☐	☒	Eukaryotic cell lines
☒	☐	Palaeontology and archaeology
☒	☐	Animals and other organisms
☒	☐	Clinical data
☒	☐	Dual use research of concern
☒	☐	Plants

Methods

n/a	Involved in the study	
☒	☐	ChIP-seq
☒	☐	Flow cytometry
☒	☐	MRI-based neuroimaging

Eukaryotic cell lines

Policy information about cell lines and Sex and Gender in Research

Cell line source(s)	HEK293T cell line used for protein expression was gifted from Dr. Torres Lab (UCLA Department of Chemistry & Biochemistry), which was originally bought from ATCC with the catalog# CRL-3216.
Authentication	None of the cell lines used were authenticated.
Mycoplasma contamination	Cell lines were not tested for mycoplasma contamination.
Commonly misidentified lines (See ICLAC register)	No commonly misidentified lines were used.

Plants

Seed stocks	No seed stock was used in our studies.
Novel plant genotypes	No novel plant genotype was produced in our studies.
Authentication	No authentication procedures for seed stocks or novel genotypes were involved in our studies.
