# Supplementary material for: Structures of human telomerase with BIBR1532 reveal novel mechanism of inhibition
Source: Nat Chem Biol. 2026 Jun 2;22(11):1854–62. doi: 10.1038/s41589-026-02238-6 (PMC13710158; doi:10.1038/s41589-026-02238-6)
Supplement: Supplementary file 3 [file 41589_2026_2238_MOESM3_ESM.pdf]

Peer Review File

Structures of human telomerase with BIBR1532 reveal novel mechanism of inhibition

Corresponding Author: Professor Juli Feigon

Version 0:

Reviewer comments:

Referee #1

(Remarks to the Author)
Wang, et al re-evaluate the mechanism of human telomerase reverse transcriptase (TERT) inactivation by a small molecule inhibitor BIBR1532 first reported 25 years ago. Although TERT has been an attractive candidate for small molecule inhibitors since its cloning, and TERT activation in cancers is firmly established, therapeutic innovation has lagged with no small molecule TERT inhibitors approved for clinical use. BIBR1532 emerged early on as an attractive lead but rational drug design based on a crystal structure with Tribolium castaneum TERT showing engagement of the C-terminal extension have not produced more effective inhibitors. In this elegant cryo-EM based study, Wang et al now convincingly reveal the BIBR1532 binding site on human TERT as the RT domain rather than the CTE. Multiple structures of a complex of TERT, TPP1, the RNA template hTER, and a DNA substrate, with and without BIBR1532 show a non-competitive mode of inhibition by binding near but not within the active site, without disrupting template-DNA engagement. Several novel interactions are mapped throughout the RT domain and validated using alanine scanning mutagenesis in direct telomerase activity assays, showing that residues engaged by BIBR1532 are important both for catalysis and also inhibition by BIBR1532. In the course of the studies, the authors observe an uncommon 'static' mechanism of catalysis by the fingers and palm of TERT relative to other polymerases, which the authors further show is due to a clamping of a finger domain by the T motif. Nucleotide engagement thus involves movement of just a few amino acid side chains more so than large-scale translation of the finger domain into the palm as seen in other RTs such as HIV RT. In this framework, the impact of BIBR1532 on sequential nucleotide addition is further scrutinized, revealing its major inhibitory impact on incorporation of the first dGTP molecule after translocation of the DNA substrate. Additional cryo-EM structures with and without a non-hydrolyzable dGTP unexpectedly show occupancy of what is supposed to represent a translocated template-DNA duplex in the active site (T0D0 position), which only moves into a confirmation permitting catalysis (T1D0 position) in the presence of the incoming nucleotide. With BIBR1532, the T1D0 state is not as easily resolved in the presence of nucleotide and proposed "intermediate" states comprise >50% of the conformations. Thus a new mechanism of inhibition is proposed wherein BIBR1532 further disrupts an inherently slow first step of nucleotide entry and template-DNA translocation.

Overall this is an outstanding study with significant and convincing mechanistic insights not only for how BIBR1532 inhibits TERT but also mechanistic aspects of TERT as a reverse transcriptase, with implications for understanding disease-associated mutations and advancing the development of therapeutic inhibitors. There are no significant flaws in this study. I have relatively minor feedback on the following points.

1) The appearance of three heterogeneous Classes using d(TTAGGG)3TTAG plus dGpNHpp in the presence of BIBR1532 indicates disruption of the incoming nucleotide bound/translocated state, but does not reveal precisely how. In the text, Lines 275-277 suggest that inhibition of translocation may be because of BIBR1532 binding key amino acids neighboring the active site and restricting protein dynamics required for duplex translocation. Lines 296-300 seem to suggest more of a steric impact on translocation by BIBR1532 ("occupies the limited free space"). Fig. 5E looks like BIBR is nestling and restricting the template (but it is actually the Finger, similarly colored). I suggest clarifying this model and wording for the reader.

2) In Fig. 3i, it could be helpful to split the panel into two – with and without nucleotide – highlighting in color the mobile side chains of only the most interesting residues – R631, Q833, Y717. This is very interesting but hard to see in the single panel

3) Line 316-320 – implies that BIBR1591 was designed via SAR studies based on TcTERT model, but this was not the case – BIBR1591 was another analog in the original paper preceding TcTERT structure-based SAR. I suggest revising this wording.

4) While this could be its own study, it would be an exciting addition if the authors wish to include other pertinent examples from two decades of BIBR SAR where key analogs' activities (perhaps those that are more potent) can also be rationalized,

as shown in Ext. Fig 8d.

5) Line 136, I believe refers to V658A rather than V638A

Referee #2

(Remarks to the Author)
This manuscript by Wang et al. describes the structure-function study of BIBR1532 inhibition of human telomerase activity. The authors used cryo-EM single particle analysis to determine several high-resolution structures of human telomerase bound to BIBR1532 at various relevant catalytic states. Control cryo-EM maps without BIBR1532 were obtained to provide rigorous, levelled comparative insights. Their structural insights and interpretation were tested and validated with mutagenesis and direct telomerase assays. Overall, this is an important body of work that should be made available to the telomere research community as soon as possible, especially to scientists developing drugs targeting telomerase. There are also novel insights into why telomerase is error-prone as compared to other replicative DNA polymerases.

I appreciate the authors and editor for making the maps and models available for review.

Before I can recommend publication, I have several comments for the authors, which I hope they can address:

Major:
1. Many claims or interpretations were about the active site or certain regions of the model looking similar with and without BIBR1532 added. A simple RMSD calculation between the models, focusing on the active or specific regions, can be easily done and validates the authors' visual inspection. For example, see page 4, lines 107-109.
2. Page 3, line 82: Can the authors clarify if the direct assay done for Fig. 1e had the reactions set up the same way as their cryo-EM samples? Page 3, lines 75-77 said BIBR1532 was added after telomerase-primer complexes were formed for cryo-EM work.
3. The authors described BIBR1532 as a non-competitive inhibitor for telomerase. The structural data supported this interpretation. However, an easy biochemical test (also feasible for the authors) to support this interpretation would be to determine whether increasing the substrate concentration (primer) can rescue telomerase activity. I assume that increasing primer concentration will not rescue telomerase activity if it is, indeed, a non-competitive inhibitor. If other papers have already shown this, please reference them.
4. Figure panels 3a-f are not visually very helpful in comparing finger domain differences. Maybe the authors can try overlaying ribbon representations instead.

Minor:
1. Page 4, lines 84-91 seem to belong to an introduction section. I would recommend moving it to that section or making it concise in the results section.
2. Page 4, line 97: "…allowed precise modeling". The authors should define what constitutes precise modeling. The local resolution? A model-to-map score for this map density would be helpful here.
3. Page 5, lines 130-131: Are these residues conserved?
4. Page 6, lines 144-145: Please briefly describe in the main text or figure legend how these inhibition levels are calculated.
5. Page 6, lines 150-152 said the mentioned residues were not conserved in Tetrahymena or Tribolium castaneum telomerase. And then the next lines said these residues are conserved in telomerase. I understand what the authors are saying, but I think this passage needs rewriting to be clearer.
6. I thought the "The rigid TERT finder explains…telomerase" section should be moved to a discussion section or the summary section. It is a fair interpretation, but, after all, still based on a comparative analysis at a qualitative level.
7. Page 8, line 203: "Upon binding of dTpNHpp to form the ternary complex.." can be rewritten.
8. Figure 4b,c quantification: I recommend the authors include details to how they quantify these % changes. Did they do it relative to total primer (% primer extended) and how they use RC for normalization?
9. Page 9, lines 235-236: "…cryo-EM maps at …., respectively". I think defining the order of the referred complexes would be helpful or clearer.
10. Page 10, lines 256: "….constitutively open nucleotide binding pocket" needs a reference to a figure or the prior result section.
11. Page 13, line 334: "do not" to "did not" seems like the correct tense.

Version 1:

Reviewer comments:

Referee #1

(Remarks to the Author)
The authors have satisfactorily addressed my critiques.

Referee #2

(Remarks to the Author)
The reviewers did a great job addressing all my concerns. I have no further concerns and believe the revised manuscript is

ready for publication now.

Open Access This Peer Review File is licensed under a Creative Commons Attribution 4.0 International License, which permits use, sharing, adaptation, distribution and reproduction in any medium or format, as long as you give appropriate credit to the original author(s) and the source, provide a link to the Creative Commons license, and indicate if changes were made.
In cases where reviewers are anonymous, credit should be given to 'Anonymous Referee' and the source.
The images or other third party material in this Peer Review File are included in the article’s Creative Commons license, unless indicated otherwise in a credit line to the material. If material is not included in the article’s Creative Commons license and your intended use is not permitted by statutory regulation or exceeds the permitted use, you will need to obtain permission directly from the copyright holder.
To view a copy of this license, visit https://creativecommons.org/licenses/by/4.0/

Response to Reviewers (in blue):
We thank the reviewers for their clear appreciation of the significance of our work, and their helpful comments to improve the manuscript. We have addressed all comments, and our detailed, point-by-point responses are provided below.

Referee #1 (Remarks to the Author):

Wang, et al re-evaluate the mechanism of human telomerase reverse transcriptase (TERT) inactivation by a small molecule inhibitor BIBR1532 first reported 25 years ago. Although TERT has been an attractive candidate for small molecule inhibitors since its cloning, and TERT activation in cancers is firmly established, therapeutic innovation has lagged with no small molecule TERT inhibitors approved for clinical use. BIBR1532 emerged early on as an attractive lead but rational drug design based on a crystal structure with Tribolium castaneum TERT showing engagement of the C-terminal extension have not produced more effective inhibitors. In this elegant cryo-EM based study, Wang et al now convincingly reveal the BIBR1532 binding site on human TERT as the RT domain rather than the CTE. Multiple structures of a complex of TERT, TPP1, the RNA template hTER, and a DNA substrate, with and without BIBR1532 show a non-competitive mode of inhibition by binding near but not within the active site, without disrupting template-DNA engagement. Several novel interactions are mapped throughout the RT domain and validated using alanine scanning mutagenesis in direct telomerase activity assays, showing that residues engaged by BIBR1532 are important both for catalysis and also inhibition by BIBR1532. In the course of the studies, the authors observe an uncommon ‘static’ mechanism of catalysis by the fingers and palm of TERT relative to other polymerases, which the authors further show is due to a clamping of a finger domain by the T motif. Nucleotide engagement thus involves movement of just a few amino acid side chains more so than large-scale translation of the finger domain into the palm as seen in other RTs such as HIV RT. In this framework, the impact of BIBR1532 on sequential nucleotide addition is further scrutinized, revealing its major inhibitory impact on incorporation of the first dGTP molecule after translocation of the DNA substrate. Additional cryo-EM structures with and without a non-hydrolyzable dGTP unexpectedly show occupancy of what is supposed to represent a translocated template-DNA duplex in the active site (T0D0 position), which only moves into a confirmation permitting catalysis (T1D0 position) in the presence of the incoming nucleotide. With BIBR1532, the T1D0 state is not as easily resolved in the presence of nucleotide and proposed “intermediate” states comprise >50% of the conformations. Thus a new mechanism of inhibition is proposed wherein BIBR1532 further disrupts an inherently slow first step of nucleotide entry and template-DNA translocation.

Overall this is an outstanding study with significant and convincing mechanistic insights not only for how BIBR1532 inhibits TERT but also mechanistic aspects of TERT as a reverse transcriptase, with implications for understanding disease-associated mutations and advancing the development of therapeutic inhibitors. There are no significant flaws in this study. I have relatively minor feedback on the following points.

1) The appearance of three heterogeneous Classes using d(TTAGGG)3TTAG plus dGpNHpp in the presence of BIBR1532 indicates disruption of the incoming nucleotide bound/translocated state, but does not reveal precisely how. In the text, Lines 275-277 suggest that inhibition of translocation may be because of BIBR1532 binding key amino acids neighboring the active site and restricting protein dynamics required for duplex translocation. Lines 296-300 seem to suggest more of a steric impact on translocation by BIBR1532 ("occupies the limited free space"). Fig. 5E looks like BIBR is nestling and restricting the template (but it is actually the Finger, similarly colored). I suggest clarifying this model and wording for the reader.

Thank you for the figure suggestion. To reduce confusion in Fig. 5e, we added clear color labels for the DNA and RNA and outlined the finger domain with a thicker border, so it is apparent that BIBR1532 sits next to (and restrains) the finger/Motif B unit rather than the template itself.
We have clarified in the section “Mechanism of inhibition by BIBR1532” that the three different conformations/Classes are intermediates in the movement of the template during each nt addition step (lines 307-311), visible for this slow step with BIBR bound. The inhibition is allosteric, i.e. BIBR1532 affects activity by binding at a site distant from the active site. In this case, we propose that when BIBR1532 is in the BIBR binding pocket it damps dynamics in the functional unit comprising the 5-stranded β-sheet (from finger, motif B, TRBD) and motif B loop that includes residues that interact with the template that have to move to the next nucleotide at each step of nt addition.

2) In Fig. 3i, it could be helpful to split the panel into two – with and without nucleotide – highlighting in color the mobile side chains of only the most interesting residues – R631, Q833, Y717. This is very interesting but hard to see in the single panel

We have split Fig. 3i into two panels (± nucleotide) and highlighted the side-chain movements of R631, Q833, and Y717 in color to improve visibility as suggested.

3) Line 316-320 – implies that BIBR1591 was designed via SAR studies based on TcTERT model, but this was not the case – BIBR1591 was another analog in the original paper preceding TcTERT structure-based SAR. I suggest revising this wording.

Thank you for catching this. Indeed, BIBR1591 was later assumed to form those additional hydrophobic interactions based on the TcTERT model (ref.17). We have re-worded this in the Summary and also earlier in the results.

4) While this could be its own study, it would be an exciting addition if the authors wish to include other pertinent examples from two decades of BIBR SAR where key analogs’ activities (perhaps those that are more potent) can also be rationalized, as shown in Ext. Fig 8d.

We reviewed the published BIBR analog literature to identify additional compounds with clearly improved potency against human telomerase that could be mapped onto our newly identified binding pocket. However, we did not find validated BIBR-family analogs that outperform BIBR1532 against human telomerase across comparable assays. Many reported analogs were developed in other medicinal-chemistry contexts (such as Thrombin Inhibitor BIBR1048) and/or were later referenced to *Tc*TERT model. We have revised the Summary to better contextualize prior SAR efforts, clarify their dependence on the *Tc*TERT-based model over the past 10 years, and explain how our structures provide a framework for future structure-guided optimization of BIBR-like inhibitors.

5) Line 136, I believe refers to V658A rather than V638A

We have corrected this typo, thank you.

Referee #2 (Remarks to the Author):

This manuscript by Wang et al. describes the structure-function study of BIBR1532 inhibition of human telomerase activity. The authors used cryo-EM single particle analysis to determine several high-resolution structures of human telomerase bound to BIBR1532 at various relevant

catalytic states. Control cryo-EM maps without BIBR1532 were obtained to provide rigorous, levelled comparative insights. Their structural insights and interpretation were tested and validated with mutagenesis and direct telomerase assays. Overall, this is an important body of work that should be made available to the telomere research community as soon as possible, especially to scientists developing drugs targeting telomerase. There are also novel insights into why telomerase is error-prone as compared to other replicative DNA polymerases.

I appreciate the authors and editor for making the maps and models available for review.

Before I can recommend publication, I have several comments for the authors, which I hope they can address:

Major:
1. Many claims or interpretations were about the active site or certain regions of the model looking similar with and without BIBR1532 added. A simple RMSD calculation between the models, focusing on the active or specific regions, can be easily done and validates the authors' visual inspection. For example, see page 4, lines 107-109.

Thank you for this helpful suggestion. We have now quantified these comparisons by calculating all-atom RMSDs for the BIBR binding site region and for the active site region between structures determined in the absence and presence of BIBR1532. These values are reported in Extended Data Table 2 and range from 0.86 to 1.22 Å, consistent with only minor conformational differences and supporting our qualitative observations. The RMSD calculation using ChimeraX is described in the Methods section.

2. Page 3, line 82: Can the authors clarify if the direct assay done for Fig. 1e had the reactions set up the same way as their cryo-EM samples? Page 3, lines 75-77 said BIBR1532 was added after telomerase-primer complexes were formed for cryo-EM work.

For cryo-EM, since BIBR1532 is a non-competitive inhibitor that does not impair DNA binding (ref. 12), we added BIBR1532 after pre-incubating telomerase with the DNA primer to form a stable telomerase–primer complex and to trap defined catalytic states for structural analysis. In contrast, for the direct activity assays, the DNA primer and BIBR1532 were added at the start of the reaction, which is the standard setup for measuring inhibition during turnover (ref. 12). In addition, the activity assays require a substantial excess of primer to ensure accurate quantification of processivity (excess primer is needed so that if synthesis terminates it re-starts with a new primer). Cryo-EM sample preparation necessarily uses much lower primer concentrations to minimize background from unbound nucleic acid and avoid obscuring particle features on the grids. We have clarified these differences in the revised Methods.

3. The authors described BIBR1532 as a non-competitive inhibitor for telomerase. The structural data supported this interpretation. However, an easy biochemical test (also feasible for the authors) to support this interpretation would be to determine whether increasing the substrate concentration (primer) can rescue telomerase activity. I assume that increasing primer concentration will not rescue telomerase activity if it is, indeed, a non-competitive inhibitor. If other papers have already shown this, please reference them.

Prior enzyme-kinetic experiments, including assays testing whether increased DNA primer can rescue activity, showed that BIBR1532 is a mixed-type non-competitive inhibitor of telomerase

(ref. 12). We have revised the text to mention those kinetic experiments found in ref 12, which rigorously showed that the inhibition was non-competitive.

4. Figure panels 3a-f are not visually very helpful in comparing finger domain differences. Maybe the authors can try overlaying ribbon representations instead.

We have revised Fig. 3a–f to improve visual comparison of the finger domain as suggested.

Minor:

1. Page 4, lines 84-91 seem to belong to an introduction section. I would recommend moving it to that section or making it concise in the results section.

We had debated about this when writing the manuscript, but this section also serves to describe the structure of telomerase–TPP1 with BIBR solved for this paper that is shown in Figure 1. After discussion again, we felt it best to leave it where it is.

2. Page 4, line 97: "…allowed precise modeling". The authors should define what constitutes precise modeling. The local resolution? A model-to-map score for this map density would be helpful here.

Thank you for the suggestion. We revised the statement to "accurate modeling" and added an objective model-to-map metric (Q-score). We calculated the Q-scores for BIBR1532 density in all 4 structures, which range from 0.613 to 0.784, and we report these values in Extended Data Fig. 3. The Q score calculation using ChimeraX is now described in the Methods section.

3. Page 5, lines 130-131: Are these residues conserved?

We showed the conservation of the BIBR1532 binding-pocket residues in ED Fig. 6. Most pocket residues are not conserved in evolutionarily distant species (e.g., Tribolium casteneum (flour beetle), insect cells Spodoptera frugiperda, ciliate Tetrahymena thermophila ; Extended Data Fig. 6b). Within vertebrates, conservation is limited, with the strongest conservation in Motif B, including Gln827, Gly830, Pro832, and Ser835.

4. Page 6, lines 144-145: Please briefly describe in the main text or figure legend how these inhibition levels are calculated.

We have added brief introduction in the figure legend (Fig. 2h and Extended Data Fig. 5f) and a more detailed description of how the inhibition levels were calculated in the Methods section.

5. Page 6, lines 150-152 said the mentioned residues were not conserved in Tetrahymena or Tribolium castaneum telomerase. And then the next lines said these residues are conserved in telomerase. I understand what the authors are saying, but I think this passage needs rewriting to be clearer.

For the second statement, we said the residues were in motifs specific to telomerase (not that they were sequence conserved). We have rewritten the text as suggested to make this clearer.

6. I thought the "The rigid TERT finder explains…telomerase" section should be moved to a discussion section or the summary section. It is a fair interpretation, but, after all, still based on a comparative analysis at a qualitative level.

We decided to keep this section in its current position because (1) it leads directly from the previous section and then to the following section, and (2) we present a detailed description of the actual sidechain movements within the nucleotide binding pocket that are present (Figure 3 i,j). We can then synthesize our observations into a mechanistic interpretation (better flow). Although interpretive, the statements are directly grounded in the structural comparisons presented in the results.

7. Page 8, line 203: "Upon binding of dTpNHpp to form the ternary complex.." can be rewritten.

We have rewritten the sentence.

8. Figure 4b,c quantification: I recommend the authors include details to how they quantify these % changes. Did they do it relative to total primer (% primer extended) and how they use RC for normalization?

We have expanded the Methods to clarify these details as requested (rather than just referring to methods in previous publications).

9. Page 9, lines 235-236: "…cryo-EM maps at …., respectively". I think defining the order of the referred complexes would be helpful or clearer.

We revised this sentence to avoid ambiguity by explicitly specifying the corresponding structures.

10. Page 10, lines 256: "….constitutively open nucleotide binding pocket" needs a reference to a figure or the prior result section.

We have added the relevant figure reference for this statement (Fig 3a, i).

11. Page 13, line 334: "do not" to "did not" seems like the correct tense.

Done, thank you.
