# Supplementary material for: Structures of human telomerase with BIBR1532 reveal novel mechanism of inhibition
Source: Nat Chem Biol. 2026 Jun 2;22(11):1854–62. doi: 10.1038/s41589-026-02238-6 (PMC13710158; doi:10.1038/s41589-026-02238-6)
Supplement: Supplementary file 4 [file 41589_2026_2238_MOESM4_ESM.pdf]

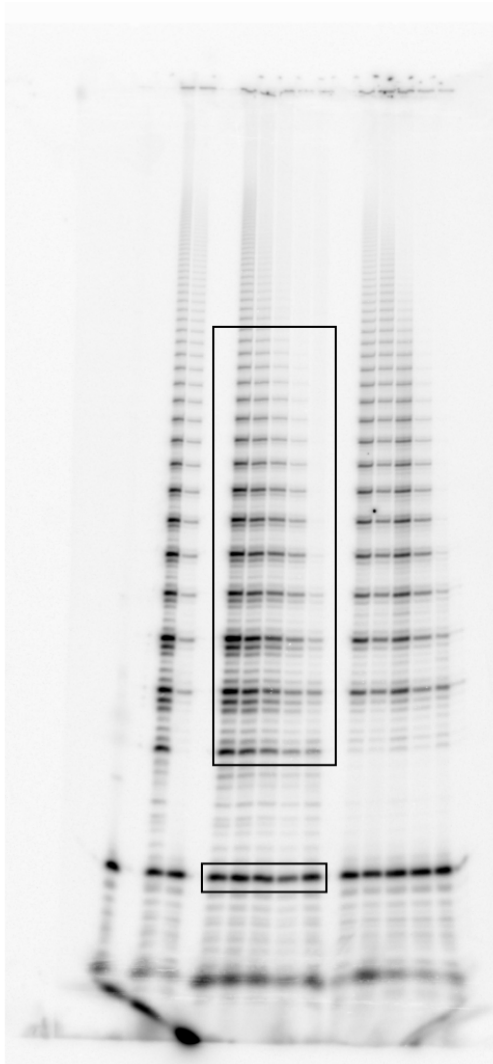

Fig. 1e

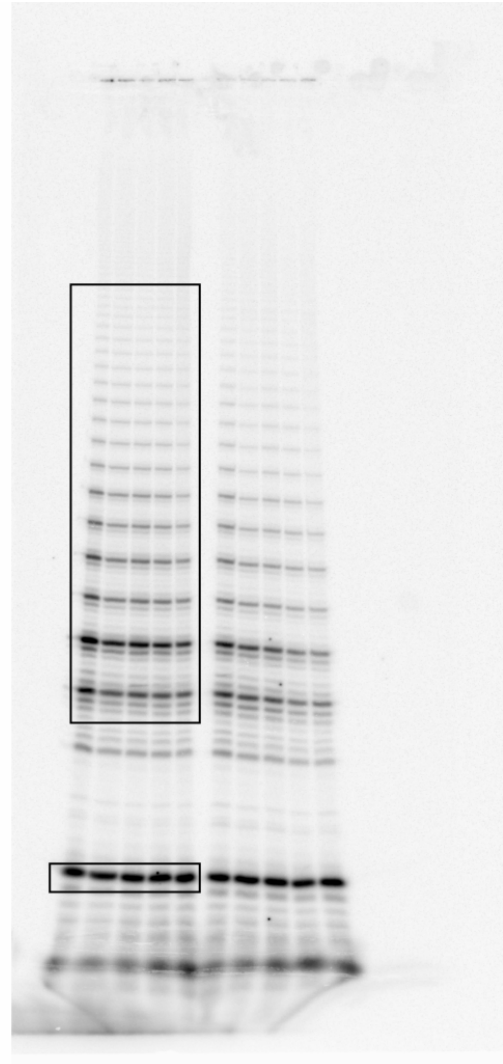

Fig. 2g_H816A

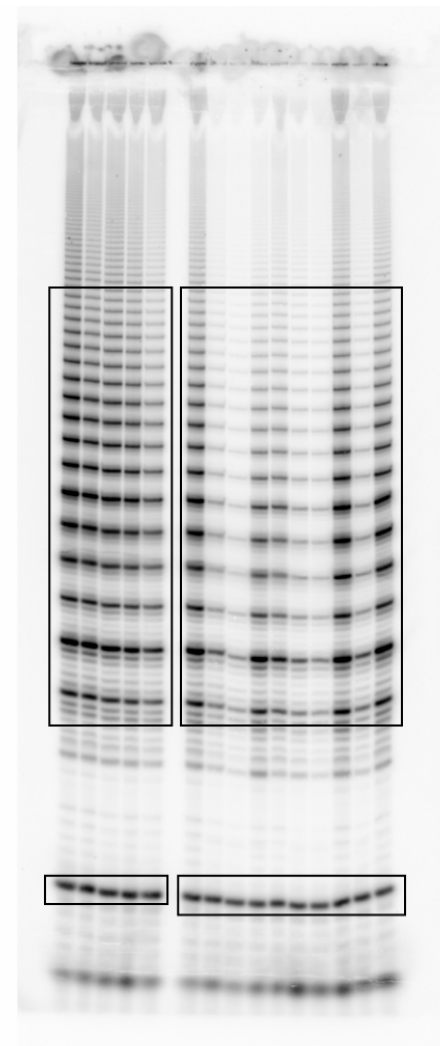

Fig. 2g_M636A ED Fig. 5c

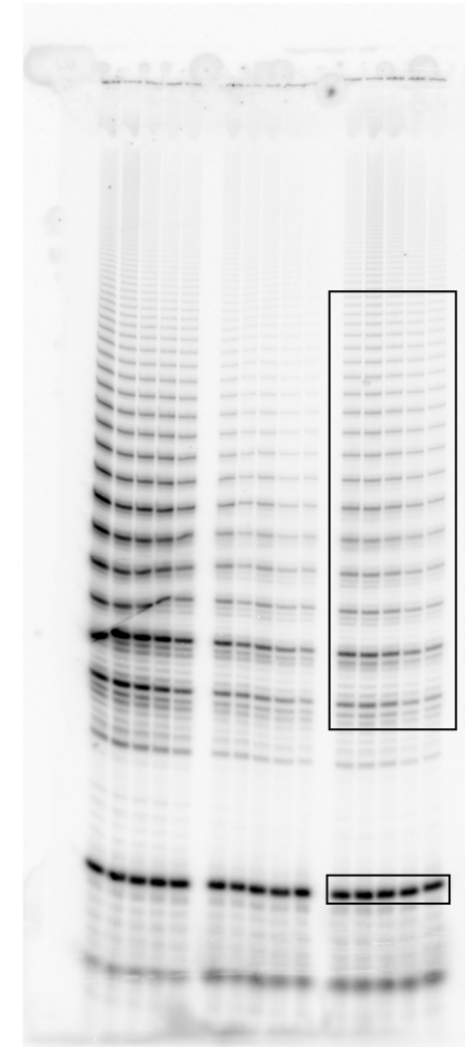

Fig. 2g_S835A

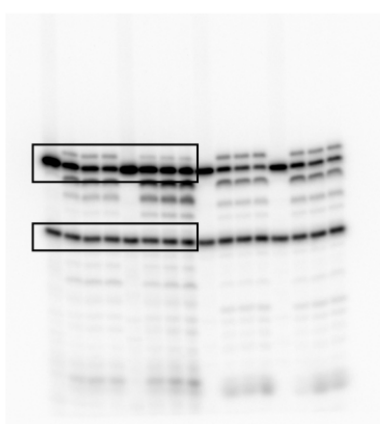

Fig. 4b_①

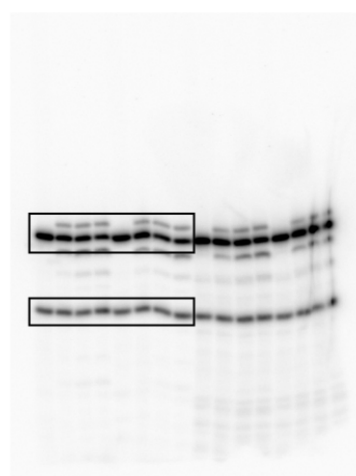

Fig. 4b_②

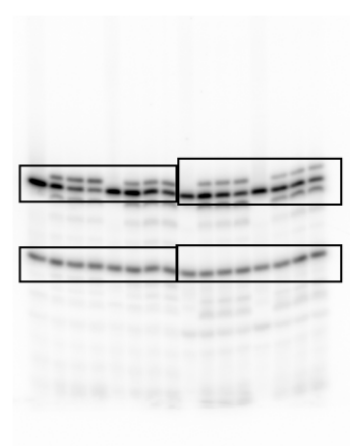

Fig. 4b_⑥ Fig. 4b_③

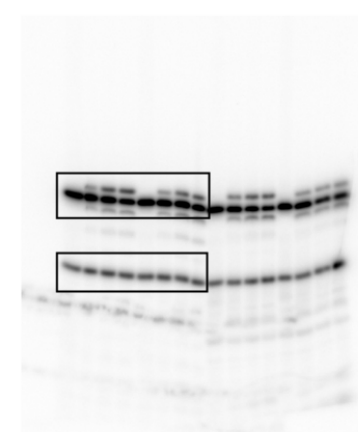

Fig. 4b_④

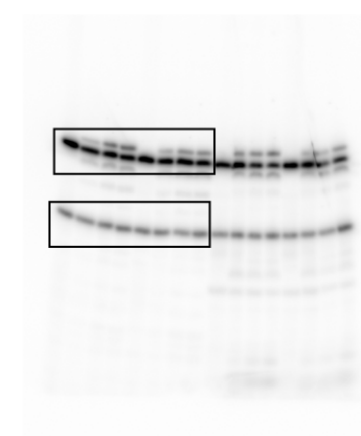

Fig. 4b_⑤

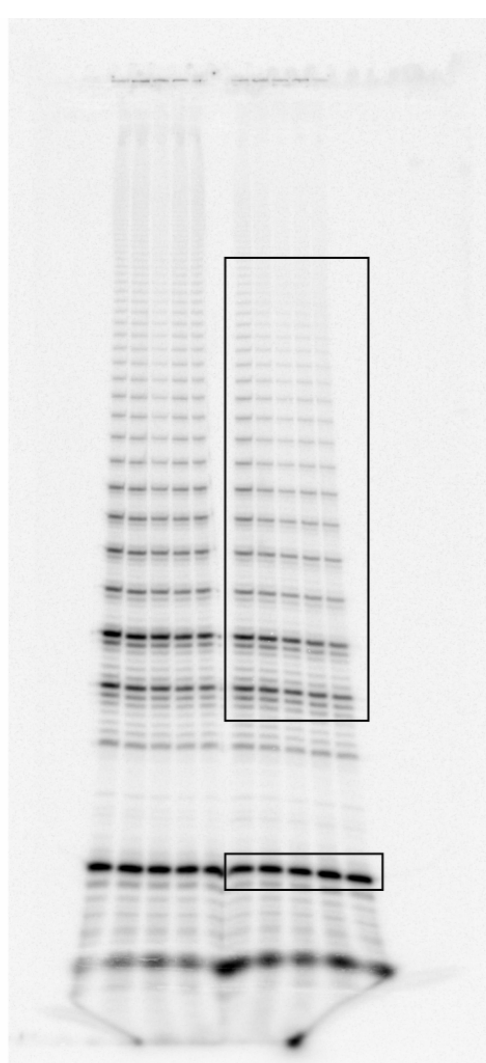

ED Fig. 5f_P832A

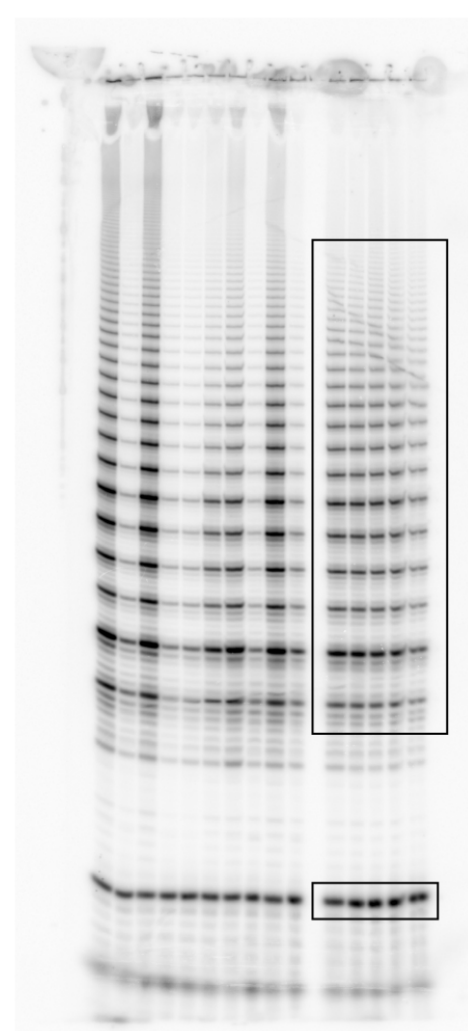

ED Fig. 5f_F812A

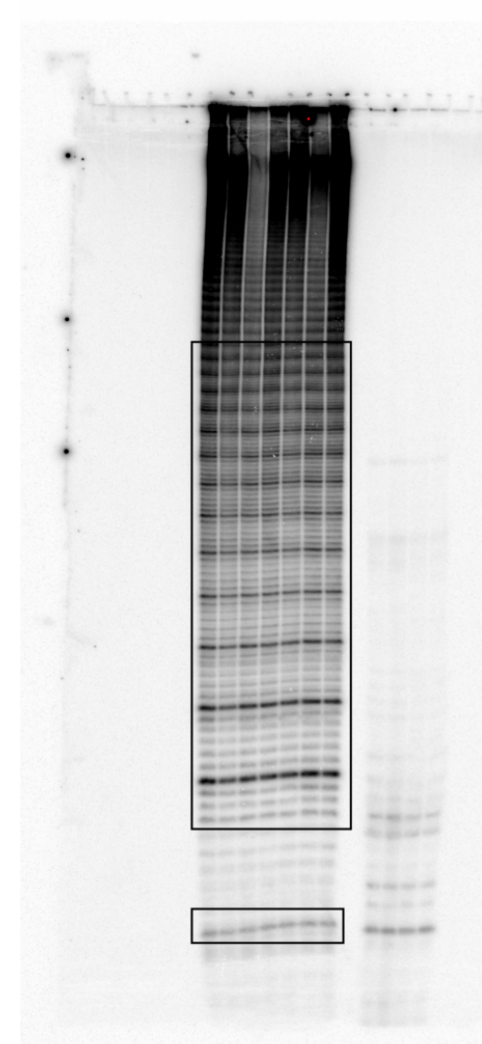

ED Fig. 6b

ED Fig. 5f_Q827A

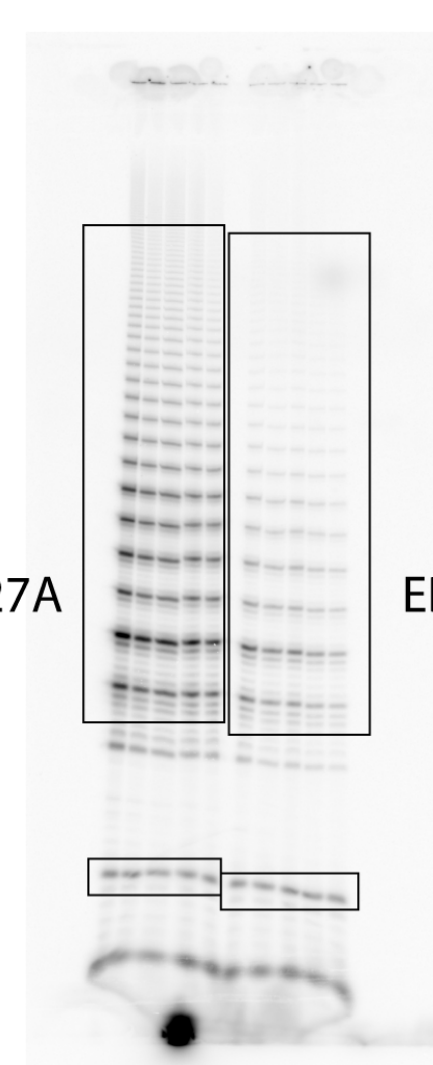

ED Fig. 5f_G830A

ED Fig. 5f_L617A

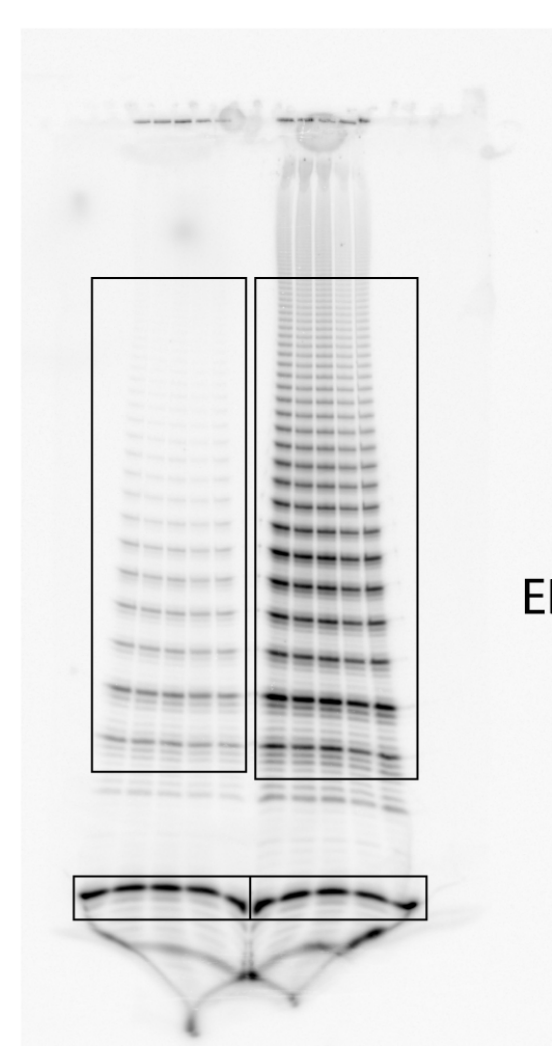

ED Fig. 5f_V658A
